# Removal of hypersignaling endosomes by simaphagy

**DOI:** 10.1080/15548627.2023.2267958

**Published:** 2023-10-16

**Authors:** Simona M. Migliano, Sebastian W. Schultz, Eva M. Wenzel, Szabolcs Takáts, Dan Liu, Silje Mørk, Kia Wee Tan, Tor Erik Rusten, Camilla Raiborg, Harald Stenmark

**Affiliations:** a Centre for Cancer Cell Reprogramming, Faculty of Medicine, University of Oslo, Oslo, Norway; b Department of Molecular Cell Biology, Institute for Cancer Research, Oslo University Hospital, Oslo, Norway; c Department of Anatomy, Cell and Developmental Biology, Eötvös Loránd University, Budapest, Hungary; d Department of Medical Cell Biology, University of Uppsala, Uppsala, Sweden

**Keywords:** Autophagy, endosome, ESCRT, receptor degradation, signaling

## Abstract

Activated transmembrane receptors continue to signal following endocytosis and are only silenced upon ESCRT-mediated internalization of the receptors into intralumenal vesicles (ILVs) of the endosomes. Accordingly, endosomes with dysfunctional receptor internalization into ILVs can cause sustained receptor signaling which has been implicated in cancer progression. Here, we describe a surveillance mechanism that allows cells to detect and clear physically intact endosomes with aberrant receptor accumulation and elevated signaling. Proximity biotinylation and proteomics analyses of ESCRT-0 defective endosomes revealed a strong enrichment of the ubiquitin-binding macroautophagy/autophagy receptors SQSTM1 and NBR1, a phenotype that was confirmed in cell culture and fly tissue. Live cell microscopy demonstrated that loss of the ESCRT-0 subunit HGS/HRS or the ESCRT-I subunit VPS37 led to high levels of ubiquitinated and phosphorylated receptors on endosomes. This was accompanied by dynamic recruitment of NBR1 and SQSTM1 as well as proteins involved in autophagy initiation and autophagosome biogenesis. Light microscopy and electron tomography revealed that endosomes with intact limiting membrane, but aberrant receptor downregulation were engulfed by phagophores. Inhibition of autophagy caused increased intra- and intercellular signaling and directed cell migration. We conclude that dysfunctional endosomes are surveyed and cleared by an autophagic process, simaphagy, which serves as a failsafe mechanism in signal termination.

**Abbreviations:** AKT: AKT serine/threonine kinase; APEX2: apurinic/apyrimidinic endodoexyribonuclease 2; ctrl: control; EEA1: early endosome antigen 1; EGF: epidermal growth factor; EGFR: epidermal growth factor receptor; ESCRT: endosomal sorting complex required for transport; GFP: green fluorescent protein; HGS/HRS: hepatocyte growth factor-regulated tyrosine kinase substrate; IF: immunofluorescence; ILV: intralumenal vesicle; KO: knockout; LIR: LC3-interacting region; LLOMe: L-leucyl-L-leucine methyl ester (hydrochloride); MAP1LC3/LC3: microtubule associated protein 1 light chain 3; MAPK1/ERK2: mitogen-activated protein kinase 1; MAPK3/ERK1: mitogen-activated protein kinase 3; NBR1: NBR1 autophagy cargo receptor; PAG10: Protein A-conjugated 10-nm gold; RB1CC1/FIP200: RB1 inducible coiled-coil 1; siRNA: small interfering RNA; SQSTM1: sequestosome 1; TUB: Tubulin; UBA: ubiquitin-associated; ULK1: unc-51 like autophagy activating kinase 1; VCL: Vinculin; VPS37: VPS37 subunit of ESCRT-I; WB: western blot; WT: wild-type.

## Introduction

Cells quickly and accurately respond to environmental signals, in part through fine-tuning the amount and localization of plasma membrane receptors. Activated cell surface receptors are typically internalized by endocytosis and transported by endosomes, which act as transient platforms for receptor signaling, degradation and recycling. Failure in trafficking or downregulation of receptors can lead to fatal pathologies [1-3].

Endosomes sequester and package receptors into intralumenal vesicles (ILVs), resulting in multivesicular endosomes, an essential process to avoid prolonged receptor signaling from endosomes. Receptor sorting and ILV formation are mediated by the endosomal sorting complex required for transport (ESCRT) machinery. ESCRT proteins are dynamically recruited from the cytosol and activated at the endosomal limiting membrane, where they mediate membrane involution into the endosomal lumen [4-6].

The ESCRT machinery consists of four multiprotein subcomplexes: ESCRT-0, -I, -II and -III, and the ATPase VPS4 [7]. These subunits remodel and seal a multitude of cellular membranes, thereby influencing cell homeostasis and membrane integrity [6, 8, 9]. On endosomes ESCRT-0 plays a central role in recognizing the ubiquitin residues of endosomal cargo, thereby mediating cargo crowding into microdomains. Along with the ubiquitin-binding ESCRT-I and -II it initiates membrane involution. ESCRT-II, in turn, is able to initiate ESCRT-III assembly. ESCRT-III polymers together with the AAA-ATPase VPS4 complete membrane deformation and the last steps of vesicle abscission into the endosomal lumen. VPS4 also mediates the disassembly of ESCRT-III filaments to allow engagement of ESCRT subunits in multiple rounds of ILV formation.

The ESCRT-0 subunit HGS/HRS (hepatocyte growth factor-regulated tyrosine kinase substrate) and ESCRT-I subunit VPS37/HCRP1 (VPS37 subunit of ESCRT-I) are of particular interest for endosomal trafficking and required for efficient receptor sequestration and degradation [5,10-12]. While HGS is only recruited to endosomes specifically mediating ILV formation, VPS37 has been implicated in ILV formation, but also autophagosome closure [10,13]. A loss of VPS37A has been shown to provoke cellular stress responses as well as increased invasion and has been associated with various cancers [14-17].

Here we have performed a proximity biotinylation-based proteomic analysis of ESCRT-dysfunctional endosomes. We discovered a cellular surveillance mechanism that is able to recognize and eliminate endosomes that are physically intact but present increased levels of active receptors. By mediating degradation of hyper-signaling endosomes, this mechanism ensures signal termination under conditions of impaired endolysosomal downregulation of signaling receptors.

## Results

### Autophagy receptors and MAP1LC3B/LC3B are recruited to endosomes with impaired ESCRT function

**Figure 1.**
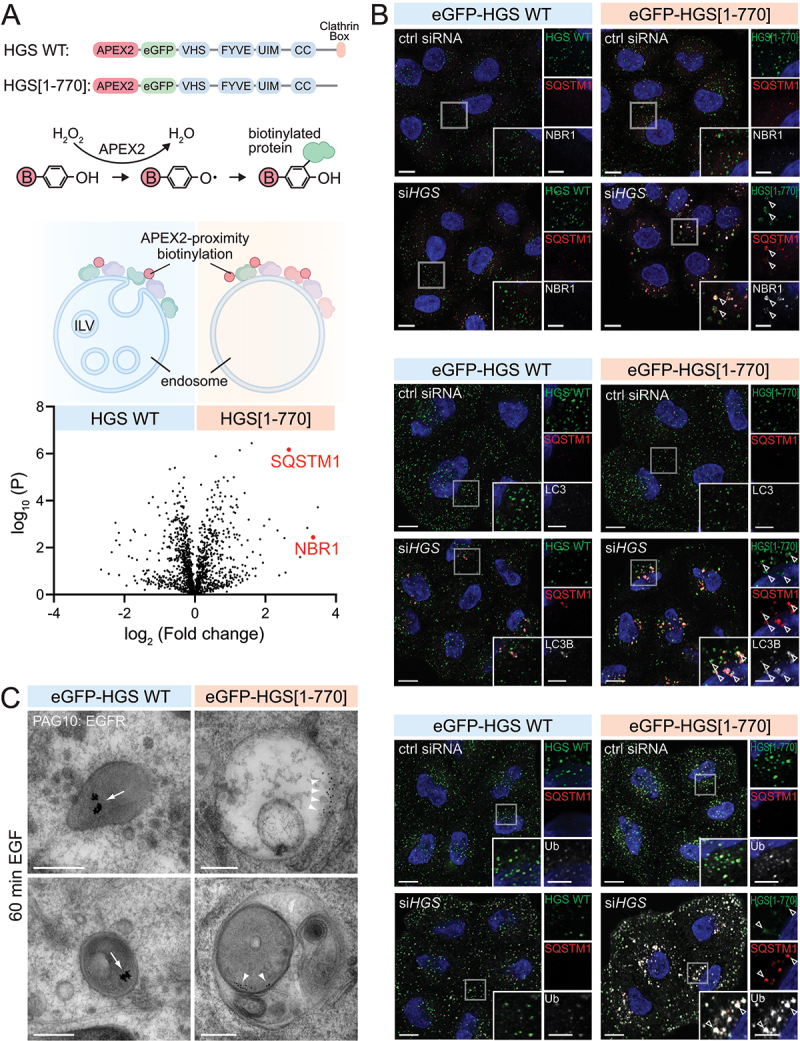
SQSTM1, NBR1 and LC3B are recruited to HGS-mutant endosomes. (a) Domain structure of APEX2-eGFP-HGS WT and APEX2-eGFP-HGS[1-770], which is missing the C-terminal clathrin box. Cell lines stably expressing APEX2-eGFP-HGS WT or -HGS[1-770] fusion proteins have been used for APEX2-proximity biotinylation of endosomally-recruited proteins. Volcano plot highlighting SQSTM1 and NBR1 enrichment in the APEX2-eGFP-HGS[1-770] mass spectrometry sample; FDR p-value 0.05. (b) Immunofluorescence staining of HGS, SQSTM1 and NBR1 in cells stably expressing eGFP-HGS WT or eGFP-HGS[1-770]. Cells are depleted for endogenous HGS or treated with control siRNA. The transgenic HGS WT and HGS[1-770] are siRNA stable. Top panel: SQSTM1 and NBR1 are recruited to HGS[1-770] endosomes (arrowheads indicating HGS, SQSTM1, NBR1 co-occurrence). Middle panel: LC3B is recruited with SQSTM1 and NBR1 to HGS[1-770] endosomes, but not in eGFP-HGS WT expressing cells (arrowheads). Bottom panel: HGS[1-770] endosomes display a strong ubiquitin (Ub) staining, which is not found in HGS WT or control cells. Scale bar: 10 µm; 5 µm for insets. (c) Representative electron micrographs of endosomes from HeLa cells stably expressing eGFP-HGS WT or HGS[1-770], depleted for endogenous HGS. Cells are stimulated 60 min with EGF to induce EGFR internalization. The 10-nm gold particles mark EGFRs. In eGFP-HGS WT cells degraded EGFR clusters in lysosomes (arrow). In eGFP-HGS[1-770] cells, EGFR is accumulating in a microdomain on the limiting membrane of endosomes (arrowheads). Bottom right panel shows an autophagosome containing a dysfunctional endosome. Scale bar: 250 nm.

In order to identify novel protein interactors on endosomes with dysfunctional ESCRT activity, we compared the proteome of functional endosomes with endosomes containing a mutant ESCRT-0 component (Fig. 1A). Specifically, we compared endosomes containing wild-type HGS versus the mutant HGS[1-770], lacking the C-terminal clathrin box, which has previously been shown to be indispensable for proper ILV biogenesis [5,18]. HGS is predominantly recruited to endosomes and does not seem required for other ESCRT driven processes, which allows us to selectively manipulate the ESCRT machinery on endosomes.

For the proteomics analysis we generated HeLa (Kyoto) cell lines stably expressing APEX2-eGFP-HGS WT or -HGS[1-770] mutant. We validated respective expression levels (Fig. S1A) and functionality by biotinylation test and immunofluorescence microscopy (Fig. S1B). Endogenous HGS was knocked down with siRNA to study the HGS[1-770] and wild-type HGS phenotypes. We detected reduced levels of clathrin subunits in the HGS[1-770] sample, which validated our APEX2-proximity labelling and semi-quantitative mass spectrometry approach. We found previously that ESCRT-0 and -I subunits persist longer on HGS[1-770] endosomes, while ESCRT-III dynamics remain unchanged [5]. These observations correspond to the distribution of ESCRT-subunits we identified in the APEX2-proximity labelling approach (Fig. S1C; left and middle panel). Unexpectedly we found the selective autophagy receptors SQSTM1/p62 (sequestosome 1) and NBR1 (NBR1 autophagy cargo receptor) to be highly enriched in the proteome of HGS[1-770] containing endosomes. We confirmed these findings with immunofluorescence microscopy and observed a strong accumulation of SQSTM1 and NBR1 surrounding HGS[1-770] endosomes. This phenotype was absent in cells expressing wild-type HGS (Fig. 1B, upper panel). Additionally, we found that LC3B, a marker of phagophore membranes, coincided with SQSTM1 and surrounded HGS[1-770] endosomes (Fig. 1B, middle panel).

HGS is the main ESCRT subunit that binds and sequesters ubiquitinated endosomal cargo into endosomal microdomains, thereby initiating ILV formation and cargo degradation [19]. As expected, we observed a strong enrichment of endosomal cargoes in the HGS[1-770] proteome compared to wild-type HGS (Fig. S1C; middle panel). In addition, immunofluorescence microscopy uncovered elevated levels of ubiquitin, which most likely represents ubiquitinated endosomal cargo on HGS[1-770] positive endosomes, but not in wild-type HGS control cells (Fig. 1B; lower panel).

### ESCRT-deficient endosomes are physically intact and sequestered by simaphagy

Accumulating endosomal cargo was also visible at the ultrastructural level when analyzing immunogold-labelled EGFR (epidermal growth factor receptor) (Fig. 1C). Protein A-conjugated gold particles of 10 nm (PAG10) were used to label cell surface EGFR (Fig. 1C; S2A, B). In late endosomes the PAG10 labelled EGFR could be seen in microdomains under the limiting membrane of endosomes [5,20]. Interestingly, within 15 min we observed electron dense areas and short double-membranes next to HGS[1-770] positive endosomes (Fig. S2B). After 60 min of EGF stimulation, the PAG10 labelled aggregates in the lumen of late endosomes/lysosomes and was never found scattered next to the limiting endosomal membrane in wild-type HGS cells. In contrast, in HGS[1-770] cells, gold labelled receptor accumulated adjacent to the limiting membrane of late endosomes in 18 of 24 observations, indicating defective internalization and receptor degradation as reported before [5]. Importantly, a subset of endosomes (3 of 24) containing EGFR at the limiting membrane were found to be engulfed, together with other organelles, in membrane-enclosed structures with characteristics of autolysosomes (Fig. 1C). This indicates that ESCRT-defective endosomes can be sequestered by autophagy. Since accumulating receptors on ESCRT-defective endosomes transmit sustained signals [19], we dub this special form of endosomal autophagy “simaphagy” (from Greek, síma = signal; phagia = eating).

Because ruptured endosomes and lysosomes can be targeted and removed by autophagy [21-26] we wondered if dysfunctional HGS could potentially lead to endosome rupture. We knocked down HGS alone or in combination with SQSTM1 and probed by immunofluorescence microscopy for localization of LGALS3 (galectin 3) and LGALS8 (galectin 8), which are markers for damaged endosomes and lysosomes [27-29]. Importantly, neither LGALS3 nor LGALS8 was recruited to HGS- and SQSTM1-depleted endosomes (Fig. S2C and S2D), indicating that the recruitment of autophagy proteins to ESCRT-dysfunctional endosomes is, unlike lysophagy, not due to membrane rupture.

### Ultrastructural analysis of simaphagy

**Figure 2.**
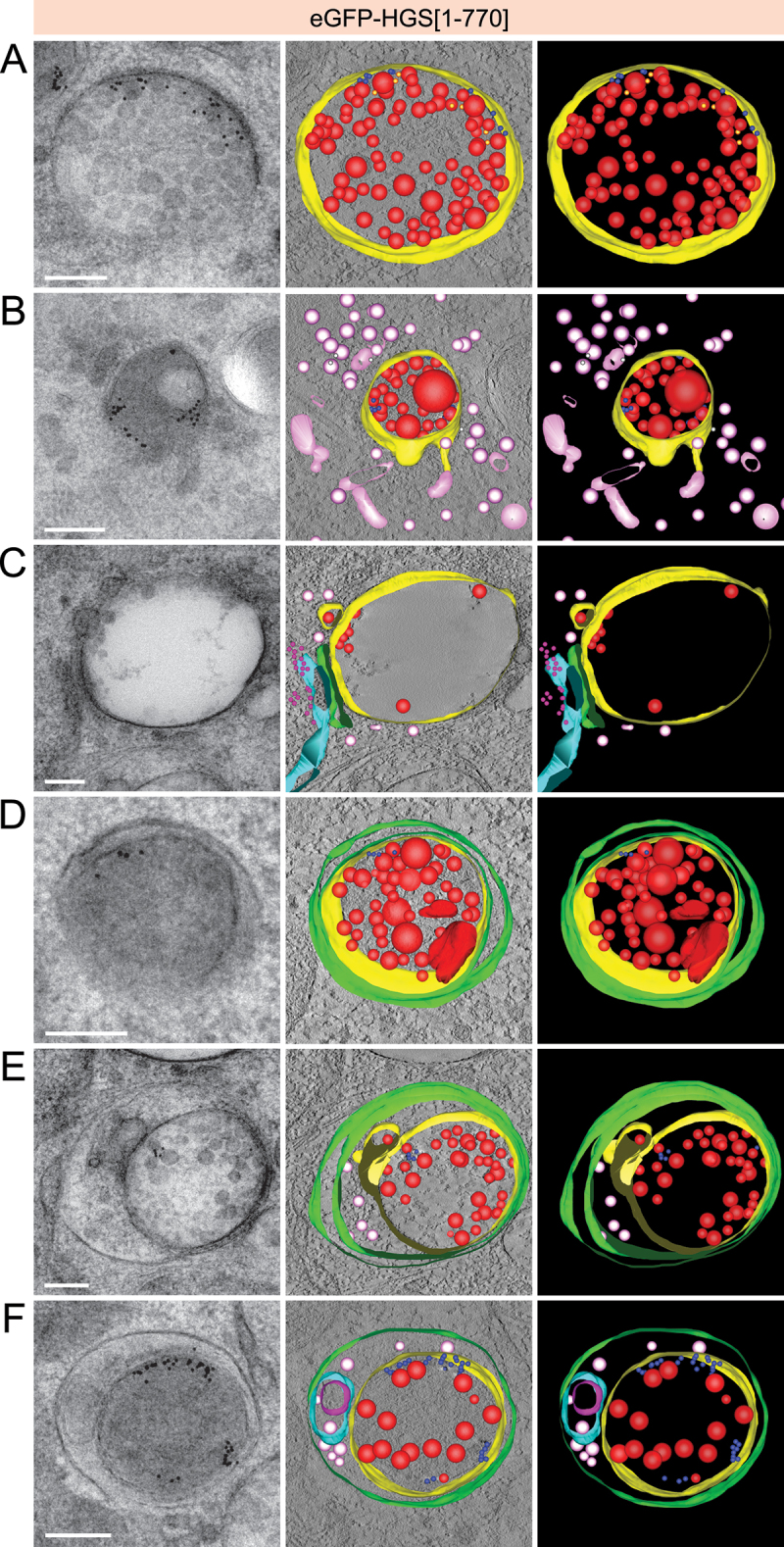
Electron tomography reveals ultrastructure of different stages of simaphagy. (a) Electron tomography projections (left) and 3D reconstructions of a representative endosome from HeLa cells with stable expression of eGFP-HGS[1-770]. Endogenous HGS has been knocked down using siRNA. Cells have been stimulated with EGF for 60 min prior to high pressure freezing and sample preparation for electron microscopy. PAG10-gold labelled EGFR [66] accumulates under the endosome limiting membrane (yellow). A subset of PAG10 [67] can be found further away from the endosome limiting membrane, most likely due to receptor-internalization as they often appear adjacent to ILVs (red). (b) Representative TEM projection and reconstruction of cytosolic vesicles (light pink) in proximity to a HGS[1-770] endosome. Often, the areas containing vesicle clusters coincide with on-section immuno-labelling of SQSTM1 (white). (c) Double-membrane sheet (green) wedged between ER (cyan; ribosomes in pink) and endosome limiting membrane (yellow). Cytosolic vesicles in close vicinity are depicted in light pink. (d) PAG10-containing endosome engulfed by a double membrane (green) in a manner resembling selective and exclusive autophagy. (e) Double membrane (green) surrounding a PAG10-containing endosome and portions of the cytosol resembling selective and non-exclusive autophagy. (f) Single membrane (green) surrounding a PAG10-containing endosome and portions of the cytosol/other organelles resembling an autolysosome in which the inner autophagic membrane has been degraded. Scale bar: 200 nm for all images.

We performed electron tomography of cells expressing HGS[1-770] in order to obtain an ultrastructural understanding of the fate of EGFRs and endosomes containing these. Samples were high-pressure frozen 60 min after EGF stimulation, and electron tomograms were taken of endosomes containing PAG10-labeled EGFR. It has been described previously that ILVs can be formed in an ESCRT-independent manner, which is in agreement with our observation that many endosomes still contained ILVs in cells expressing HGS[1-770], especially small ILVs of about 25 nm [5,30,31]. However, 60 min after EGF stimulation, 89% of the observed 91 endosomes still contained PAG10-EGFR adjacent to the limiting membrane of the endosome. In 26 of the 91 endosomes we also found PAG10-EGFR that was not adjacent to the limiting membrane but internalized into the endosome (Fig. 2A). 85% of all observed endosomes were in contact with the cytosol and not completely surrounded by a double membrane. However, more than half of these endosomes (55%) were found in close apposition to 3 or more vesicles (Fig. 2B; S3A, B).

We also detected SQSTM1 in electron tomograms. On-section immuno-gold labelling of SQSTM1 was found in close proximity to 16 of 60 PAG10-positive endosomes. Sites of SQSTM1-detection coincided with clusters of 40-60 nm wide cytosolic vesicles below in the section in 69% of the times (11 of 16 endosomes) (light pink vesicles; Fig. 2B; S3A, B).

Approximately 16% (12 of 77) of non-engulfed endosomes had one or several double membranes with varying diameter in proximity. These double membranes were of various origin: the ER network, flattened vesicles with different sizes or a combination thereof, where a double membrane sheet ran in parallel between the endosome and the ER (Fig. 2C; S3A, C). 14 of the 91 observed endosomes were completely surrounded by a double membrane (autophagosome) or engulfed into a single-membrane autolysosome (Fig. 2D-F; S3D). Taken together, the electron tomography data show that ESCRT-defective endosomes can be engulfed by double-membrane autophagosomes, providing an ultrastructural demonstration of simaphagy.

### Dynamics and morphology of simaphagy

**Figure 3.**
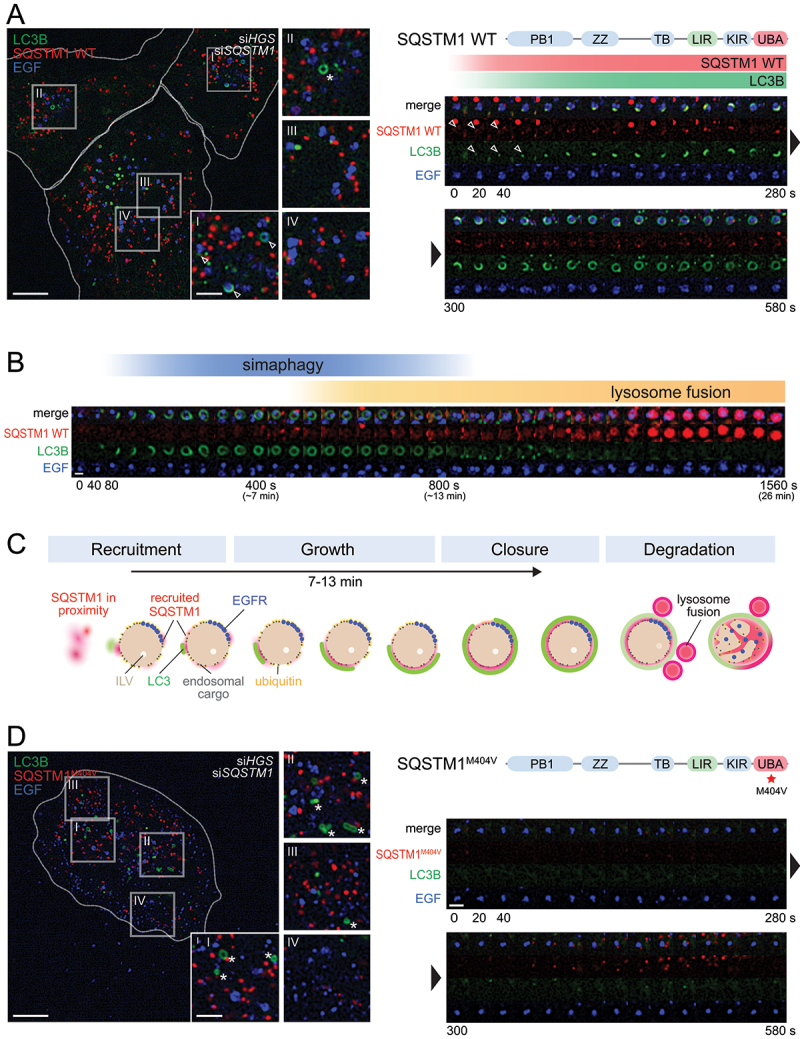
Dynamic recruitment of SQSTM1 and LC3B allows characterization of simaphagy events. (a) Left: Movie stills from live-cell imaging experiments of RPE-1 cells stably expressing GFP-LC3B and siRNA resistant mCherry-SQSTM1 WT. Endogenous HGS and SQSTM1 have been knocked down by siRNAs. Endosomes are labelled by a 2 min pulse with EGF-Alexa Fluor 647 [66]. Insets I-IV show simaphagy events in different stages (arrowhead). Phagophores not containing endosomes are marked by asterisks. Right: WT mCherry-SQSTM1 domain structure and schematic recruitment dynamics of mCherry-SQSTM1 WT and LC3B (red and green gradient). A timeline of movie stills from a representative simaphagy event is shown below. Cell outlines shown by dotted line. Scale bar: 10 µm; 3 µm for insets I-IV; 2 µm for timeline. (b) Representative timeline of a complete simaphagy event from RPE-1 mCherry-SQSTM1 WT, GFP-LC3B cells (with HGS, SQSTM1 knockdown). Blue gradient showing the approximate duration of the simaphagy event: mCherry-SQSTM1 WT and GFP-LC3B recruitment, up to eventual phagophore closure (at around 7-13 min). Decrease of GFP-LC3B signal from 10-13 min onwards. Yellow gradient showing the timeframe for lysosome recruitment and fusion. Lysosomes are indirectly visualized by mCherry-SQSTM1 fluorescence. While SQSTM1 is degraded in lysosomes, the mCherry protein remains stable and fluorescent [68,69], thus lysosomes become visible. Scale bar: 2 µm. (c) Schematic overview on the different phases in simaphagy. Dysfunctional endosomes generate few intralumenal vesicles (ILV) and accumulate endosomal cargo on the surface. EGFR frequently accumulates in a microdomain. Recruitment phase: SQSTM1 has been found in proximity to dysfunctional endosomes and surrounding dysfunctional endosomes. The recruitment of LC3B can be observed early together with SQSTM1 and marks autophagic membranes. Growth phase: the phagophore extends around the endosome. Closure takes place at around 7-13 min. The LC3B signal has becomes weaker after phagophore closure. Degradation phase: Lysosomes start to attach and fuse to finally degrade the autophagosomal content. (d) Left: Movie stills from live-cell imaging of RPE-1 cells stably expressing GFP-LC3B and mCherry-SQSTM1^M4^°^4V^. Insets highlight non-endosome containing phagophores (asterisk). Right: Domain structure of SQSTM1 with point mutation M404V. Below, timeline of a representative endosome shows no mCherry-SQSTM1^M4^°^4V^ or GFP-LC3B recruitment. Cell outlines shown by dotted line. Scale bar: 10 µm; 3 µm for insets I-IV; 2 µm for timeline.

To investigate whether the recruitment of autophagy receptors to HGS-defective endosomes resulted in their autophagic sequestration, we used hTERT-RPE-1 cells stably expressing siRNA-resistant mCherry-SQSTM1 WT and GFP-LC3B and studied these by live-cell imaging (Fig. 3). Cell lines with close to endogenous expression levels of the respective transgenes were used for all experiments (Fig. S4A). We induced dysfunctional endosomes by knockdown of endogenous HGS and studied mCherry-SQSTM1 and GFP-LC3B recruitment. Cells were pulsed with EGF-Alexa647 to label EGFR on the cell surface and the signal was monitored by live cell imaging. This enabled us to follow EGFR internalization and trafficking through the endosomal system in real time [5]. Similarly, to what was observed by immunofluorescence microscopy of fixed cells (Fig. 1B), cells with double knockdown of endogenous HGS and SQSTM1 showed an increased number of mCherry-SQSTM1- and GFP-LC3B-positive structures. A pool of these mCherry-SQSTM1 WT and GFP-LC3B signals coincided with EGF-Alexa Fluor 647-positive endosomes (Fig. 3A).

We manually tracked a total of 96 endosomes with clear mCherry-SQSTM1 WT and GFP-LC3B recruitment (representative track in Fig. 3A; Movie 1). Due to endosome movement, not all simaphagy events could be captured from start to end. However, 38% of the tracked endosomes were classified as complete simaphagy events, including: (1) a recruitment phase of SQSTM1 and LC3B, (2) a growth phase, with an extension of the phagophore, (3) a phagophore closure and (4) an onset of lysosome fusion and degradation (Fig. 3B, C).

The recruitment of mCherry-SQSTM1 WT and GFP-LC3B to EGF-labelled endosomes started with a dim signal in proximity to the EGF-labelled endosome (Fig. S4B). Within minutes mCherry-SQSTM1 WT accumulated around the endosome, sometimes forming brighter subdomains (Fig. S4B). mCherry-SQSTM1 WT and GFP-LC3B were often recruited at similar times to endosomes, together initiating simaphagy (Fig. 3A, B, C).

To study the growth and extension phase of the phagophore, we monitored GFP-LC3B, which in its lipidated form is an integral part of autophagic membranes (Fig. 3A, S4B; Movie 6). We observed that GFP-LC3B only nucleated at a single site, from where the LC3B-signal expanded around the endosomes. In a few simaphagy events we also observed hook-shaped extensions of LC3B-positive phagophores, predominantly during growth/elongation phase. This could possibly be an extension to the ER-network and provide phagophore membrane (Fig. S4C).

A clear timepoint of phagophore closure could not be determined by this imaging method. However, in a few events we observed a morphological change, where elliptical phagophores became circular. This rounding was frequently observed at 7-8 min after initiation and was likely to coincide with phagophore closure [32]. The end of a simaphagy event was typically characterized by a decrease in GFP-LC3B signal between 7-13 min after the onset and was followed by docking and fusion of lysosomes (Fig. 3B, C; S4D; Movie 5).

In an imaging period of 30 min we typically counted 2-9 simaphagy events per cell. Overall, 80-90% of such events could be found in the perinuclear region, while only a small portion was observed in the cell periphery (Fig. S4E). This suggests that only a subpopulation of endosomes is targeted by autophagy.

### SQSTM1 recruitment and subsequent phagophore formation depend on ubiquitinated endosomal cargo

Next, we aimed to investigate the recruiting signal for the autophagic receptors. Cargoes for selective autophagy are frequently labelled by ubiquitin or other “eat me” signals and recognized by autophagic receptors [33-37]. Since SQSTM1 contains a ubiquitin associated (UBA) domain we tested whether the accumulation of ubiquitinated receptors on the endosomal surface could trigger SQSTM1 recruitment.

We imaged cells stably expressing GFP-LC3B and mCherry-SQSTM1^M4^°^4V^, carrying a point mutation in the UBA domain that abolishes the capability of SQSTM1 to bind ubiquitin. We tracked a total of 81 endosomes from 8 cells and found that 75 of these did not recruit mCherry-SQSTM1^M4^°^4V^ to EGF-positive endosomes and consequently no GFP-LC3B-positive phagophores were formed (Fig. 3D; Movie 2). This suggests that ubiquitination of the endosomal cargo is indeed the recruiting signal for SQSTM1 and downstream autophagic events.

### NBR1 and SQSTM1 play overlapping roles in simaphagy

NBR1 has been described as an evolutionarily related autophagy receptor with partly overlapping functions to SQSTM1 [33]. Since we found SQSTM1 and NBR1 in the proteomic dataset, we investigated if recruitment of LC3B-positive phagophores was dependent on interaction with SQSTM1 only, or whether SQSTM1 and NBR1 play redundant roles during simaphagy. We took advantage of SQSTM1ΔLIR, a mutant lacking the LC3-interacting region (LIR) and therefore unable to recruit LC3B and autophagic membranes (Fig. S5A) [38,39]. We performed live cell imaging of mCherry-SQSTM1ΔLIR and GFP-LC3B expressing cells and tracked 80 endosomes. 72 of these endosomes showed a strong mCherry-SQSTM1ΔLIR accumulation, but only on 26 endosomes we also observed GFP-LC3B signal. Once GFP-LC3B-positive membranes were present, they seemed to heavily deform (Fig. S5B; Movie 3). Further we noticed a substantial delay in phagophore formation, where the first GFP-LC3B-positive membranes accumulated between 1-20 min after the mCherry-SQSTM1ΔLIR had appeared.

Only in 13% of all tracks we observed successfully formed phagophores (recruitment, growth and closure phase), few of these were docking to and fusing with lysosomes. This could indicate that simaphagy is impaired, but not completely abolished by the lack of SQSTM1-LC3B interaction.

To assess a potential overlapping role of the LIR-containing autophagic receptor NBR1 in simaphagy, we knocked down endogenous NBR1 in mCherry-SQSTM1ΔLIR and GFP-LC3B expressing cells (Fig. S5A, C). We manually tracked 23 endosomes and found mCherry-SQSTM1ΔLIR to be recruited normally. However, the amount of recruited GFP-LC3B to endosomes was reduced in NBR1 depleted cells. From the 23 mCherry-SQSTM1ΔLIR endosomes only 6 also recruited GFP-LC3B (Fig. S5C). This suggests that both SQSTM1 and NBR1 play a role as autophagy receptors by recruiting LC3B-positive autophagic membranes during simaphagy.

### The autophagy initiation complex is recruited to dysfunctional endosomes

**Figure 4.**
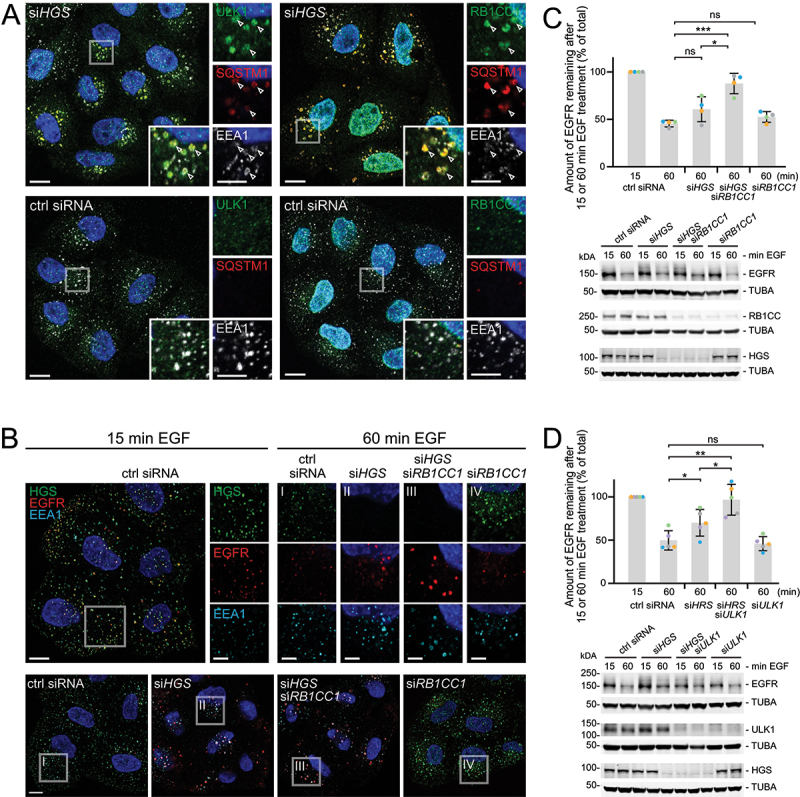
Additive inhibition of EGFR degradation by HGS depletion and autophagy inhibition. (a) ULK1 and RB1CC1 support the initiation of simaphagy events. Representative immunofluorescence staining of HeLa cells after knockdown using si*HGS* or control siRNA. RB1CC1 and ULK1 are both recruited to EEA1 and SQSTM1 positive endosomes. Arrowheads highlight the localization of RB1CC1, ULK1 and SQSTM1 on EEA1 endosomes. Scale bar: 10 µm; 5 µm for insets. (b) Representative immunofluorescence staining of HeLa cells after 15 or 60 min EGF (50 ng/ml) stimulation. HGS and RB1CC1 are knocked down by siRNA and endosomes are stained for EEA1, HGS and EGFR. The EGFR appears to accumulate in si*HGS* and even stronger in si*HGS*, si*RB1CC1* cells. Scale bar: 10 µm; 5 µm for insets I-IV. (c) Quantitative western blot analysis of undegraded EGFR, after 15 or 60 min EGF stimulation. Degradation is severely impaired upon HGS and RB1CC1 depletion. Below: Representative western blot of whole cell lysates showing HGS and RB1CC1 knockdown and EGFR protein levels. Values are normalized to 15 min control siRNA. Mean ± SD of three independent experiments. One-way ANOVA *p < 0.1, ***p < 0.001, ns = not statistically significant. (d) Quantitative western blot analysis of HeLa cells after HGS and ULK1 knockdown. Representative western blot showing protein levels and undegraded EGFR, after 15 or 60 min EGF stimulation. The quantification shows an impaired EGFR degradation after HGS and ULK1 double-knockdown. Mean ± SD of five independent experiments. Values are normalized to 15 min control siRNA. One-way ANOVA *p < 0.1, **p < 0.01, ns = not statistically significant.

The protein kinase ULK1 (unc-51 like autophagy activating kinase 1) is found together with RB1CC1/FIP200 (RB1 inducible coiled-coil 1), ATG13 and ATG101 in an initiation complex that mediates the first steps of autophagy [40,41]. RB1CC1 has been shown to interact with the endosomal protein RABEP1/Rabaptin 5 on damaged early endosomes [24]. We therefore tested if components of the autophagy initiation complex were detectable also on unruptured but HGS-depleted endosomes at early stages of simaphagy. ULK1 or RB1CC1 were not detected on endosomes in control cells. In contrast, immunofluorescence staining revealed that both ULK1 and RB1CC1 localized to endosomes and partially overlapped with SQSTM1 in HGS-depleted HeLa cells (Fig. 4A). This underlines that the autophagy initiation machinery upstream of SQSTM1, NBR1 and LC3B is involved in simaphagy.

### Simaphagy aids in EGFR degradation from dysfunctional endosomes

Next, we aimed to investigate whether simaphagy affects receptor degradation. To test this, we monitored EGFR as endosomal cargo and performed EGF-pulse-chase experiments using HeLa cells. We compared the EGFR degradation rates in cells with and without autophagy inhibition.

We stained internalized EGFR on endosomes using immunofluorescence microscopy (Fig. 4B) and quantified EGFR degradation by western blot analysis (Fig. 4C, D). After 15 min of EGF stimulation most of the surface EGFR had been internalized and localized on EEA1 positive endosomes in control cells (Fig. 4B). Sixty min after EGF stimulation, we observed a significant decrease in EGFR fluorescence intensity in control cells (Fig. 4B). This was also reflected by western blot quantifications of residual EGFR (Fig. 4C, D). Upon HGS depletion, EGFR degradation was slowed down in accordance with published data [5,20]. The combination of HGS depletion and autophagy inhibition by RB1CC1 knockdown significantly reduced EGFR degradation, causing a strong accumulation of EGFR positive endosomes in immunofluorescence microscopy (Fig. 4B). In these cells we observed an almost complete block of EGFR degradation by western blotting (Fig. 4C). A knockdown of RB1CC1 by itself did not affect EGFR degradation (Fig. 4B, C). Similar findings were obtained by knocking down HGS in combination with ULK1 (Fig. 4D). These findings highlight that simaphagy plays a role in the clearance of dysfunctional endosomes and thus aids in receptor degradation.

### Sustained EGFR activation and intercellular signaling from cells without simaphagy

The proximity biotinylation proteomics showed that dysfunctional endosomes accumulate endosomal cargo on the limiting membrane, including cell surface receptors, such as receptor tyrosine kinases (EGFR), cytokine receptors (OMSR, LIFR, IL6ST) and integrins involved in cell migration (Fig. S1C). We hypothesized that such receptors might show sustained activation (phosphorylation) and signaling in the absence of simaphagy.

**Figure 5.**
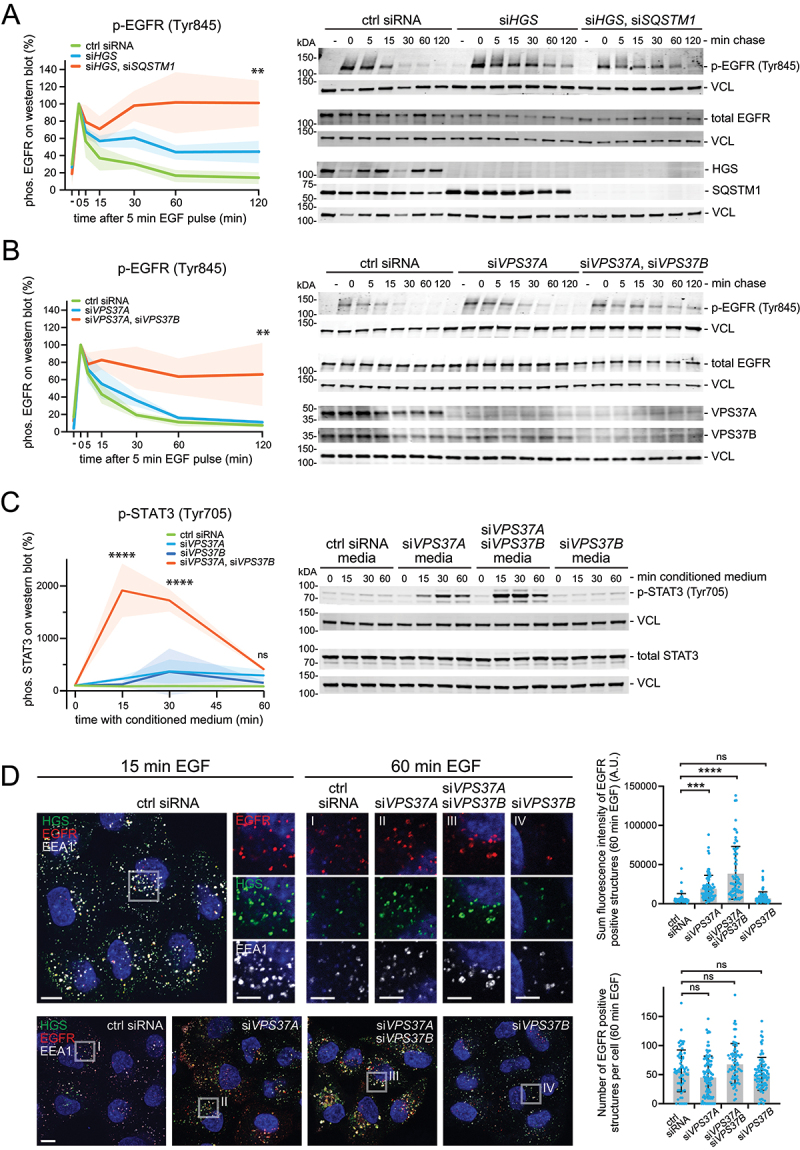
Endosomes with knockdown of VPS37A and VPS37B accumulate phosphorylated EGFR and STAT3. (a) Quantitative western blot analysis of EGFR in HeLa cells with knockdown of HGS, or HGS and SQSTM1 using siRNA. Cells were serum starved overnight and stimulated for 5 min with 5 ng/ml EGF. Knockdown of HGS prolongs the activation of EGFR (Tyr845) phosphorylation. Cells with knockdown of HGS and SQSTM1 show even stronger EGFR (Tyr845) phosphorylation over 120 min. A representative western blot for EGFR (Tyr845) is shown and the phosphorylation of downstream factors, probed on the same membrane, are shown in Figure S6A. Total EGFR levels are shown for comparison. Quantifications of EGFR (Tyr845) amount on western blot are normalized to the loading control and shown in percent. t = 0 min chase was set to 100%; results are mean ± SD of three independent experiments. Mean ± SD One-way ANOVA **p < 0.01 of timepoint 120 min. (b) Quantitative western blot analysis of EGFR (Tyr845) in HeLa cells with knockdown of VPS37A, or VPS37A and VPS37B using siRNA. Cells were serum starved overnight and stimulated for 5 min with 5 ng/ml EGF. Knockdown of VPS37A only is not sufficient to sustain prolonged EGFR (Tyr845) phosphorylation. Cells with knockdown of VPS37A and VPS37B show elevated EGFR (Tyr845) phosphorylation over 120 min. A representative western blot for EGFR (Tyr845) is shown and the phosphorylation of downstream factors, probed on the same membrane, are shown in **Figure S6C**. Total EGFR levels are shown for comparison. Quantifications of EGFR (Tyr845) amount on western blot are normalized to the loading control and shown in percent. t = 0 min chase was set to 100%; results are mean ± SD of three independent experiments. Mean ± SD One-way ANOVA **p < 0.01 of timepoint 120 min. (c) Quantitative western blot analysis of pSTAT3 (Tyr705) in HeLa cells, after incubation with conditioned medium (0, 15, 30,60 min). The conditioned medium was harvested from HeLa cells with knockdown of VPS37A or VPS37B or double knockdown of VPS37A and VPS37B. A representative western blot showing pSTAT3 (Tyr705) and total STAT3 expression are shown. Quantifications of pSTAT3 (Tyr705) are normalized to the loading control and shown in percent. Results are mean ± SD of three independent experiments. Mean ± SD, One-way ANOVA of timepoint 120 min, ****p <0.0001, ns = not statistically significant. (d) Representative immunofluorescence staining after 15 and 60 min EGF stimulation. EGFR degradation is impaired upon VPS37A and VPS37B knockdown. Insets show EGFR localization to endosomal markers HGS and EEA1. Scale bar: 10 µm; 5 µm for insets I-IV. Quantitative analysis of number and sum fluorescence intensity of EGFR positive structures in the VPS37A and VPS37B knockdown cells. The number of EGFR structures remains unaltered in all samples. The sum fluorescence intensity of EGFR accumulations is significantly increased in cells depleted for VPS37A only and with co-depletion of VPS37B. Quantification from one staining shown, observed in other independent experiments. Quantification of 50-70 cells per condition. Mean ± SD One-way ANOVA ***p < 0.001, ****p <0.0001, ns = not statistically significant.

To test this, we stimulated HeLa cells with EGF and measured EGFR phosphorylation and downstream activation of AKT and MAPK1/ERK2-MAPK3/ERK1 after different time points. We then investigated if HGS and SQSTM1 knockdown had an effect on EGF-mediated signaling. In control cells, phosphorylated EGFR (Tyr845) levels reached the maximum 5 min after the EGF pulse and were reduced to initial levels (16.7%; SD 7.0%) within 60 min (Fig. 5A). This finding was in accordance with the EGFR degradation quantifications (Fig. 4C, D). Knockdown of HGS led to prolonged EGFR (Tyr845) phosphorylation. 120 min after the EGF pulse 44.5% (SD 12.9%) of the EGFR (Tyr845) was still detectable. Interestingly, cells with double knockdown of HGS and SQSTM1 retained even higher EGFR (Tyr845) levels (101.8%; SD 35.6% after 60 min and 101.2%, SD 26.4% after 120 min) (Fig. 5A).

Downstream phosphorylation of AKT (Ser437) and MAPK1-MAPK3 (Thr202/Tyr204) was monitored in the same way. We observed a lower initial activation of AKT (Ser437) and MAPK1-MAPK3 (Thr202/Tyr204) in cells with co-depletion when compared to control cells (0-15 min chase). This is in line with the lower initial EGFR (Tyr845) levels and the previously described intricate connection between EGFR levels and numbers of autophagic structures influencing downstream signaling [42]. In control cells AKT (Ser437) and MAPK1-MAPK3 (Thr202/Tyr204) levels went to base levels after 120 min but remained elevated in HGS depleted cells (AKT (Ser437): 100.1%; SD 37.0% and MAPK1-MAPK3 (Thr202/Tyr204): 27.1%; SD 6.1%). Although we observed a high variability between the experiments, there was a trend that co-depletion of HGS and SQSTM1 enhanced the effect of prolonged substrate phosphorylation (AKT (Ser437): 137.5%; SD 59.8%; MAPK1-MAPK3 (Thr202/Tyr204): 48.5%; SD 36.6%) (Fig. S6A). Taken together, our results indicate that simaphagy contributes to the removal of dysfunctional endosomes and thereby aids with silencing of endosomal receptors and their corresponding signaling pathways.

### VPS37A VPS37B depletion perturbs EGFR downregulation and causes sustained signaling

Next, we aimed to test the effects of other essential ESCRT subunits. We were particularly interested in the ESCRT-I subunit VPS37. Mammalian cells express four isoforms of VPS37 (A-D), all containing a mod(r) region, which interacts with the ESCRT-I subunit TSG101. VPS37B, C and D contain proline-rich regions of varying length (69–193 amino acids). Only VPS37B additionally contains a PTAP motif, which mediates interaction with TSG101. Due to these differences in domain structure, all isoforms have been shown to bind with variable affinity to other ESCRT-I subunits [10,43,44]. Interestingly, VPS37A is the only isoform that harbors a ubiquitin-interacting UEV-domain and is also the only VPS37 isoform that is essential for phagophore closure in autophagy [13]. Thus, depletion of VPS37A affects both ILV formation and autophagy at the same time. We therefore hypothesized that the lack of VPS37A might cause stalled simaphagy events.

To investigate this, we conducted signaling experiments as described above. Surprisingly, EGFR (Tyr845) phosphorylation returned equally fast to basal levels in VPS37A knockdown cells as in control cells. Instead, we found that a double knockdown of VPS37A and VPS37B was required to prolong EGFR (Tyr845) phosphorylation (66.0%, +/- 36.1% after 120 min) (Fig. 5B). Similar results were observed for AKT (Ser437) and MAPK1-MAPK3 (Thr202/Tyr204). While depletion of VPS37A alone was without effect, additional knockdown of VPS37B led to an elevated phosphorylation of both downstream factors. Despite the variability between experiments a trend for prolonged AKT (Ser437) and MAPK1-MAPK3 (Thr202/Tyr204) could be detected (Fig. S6A, C).

In line with prolonged EGFR (Tyr845) phosphorylation, we expected a slower EGFR degradation upon VPS37A and VPS37B knockdown. To test this, we knocked down VPS37A and VPS37B and stained for EGFR after 15 and 60 min of EGF stimulation, followed by fluorescence microscopy. The overall number of EGFR-positive vesicles did not vary significantly between the treatments. VPS37A knockdown alone led to increased EGFR levels on EEA1 positive endosomes compared to control cells (Fig. 5D; S6D). Cells with knockdown of VPS37B only degraded EGFR normally. However, EGFR accumulation was further increased upon double knockdown of VPS37A and VPS37B. From this we conclude that VPS37A and VPS37B function partially redundantly in endosomal downregulation of EGFRs in the cell system used.

Since we found several cytokine receptors enriched on endosomes upon ESCRT inactivation by the proximity biotinylation proteomics (Fig. S1C), we asked whether cells with depletion of VPS3A and VPS37B display increased intercellular cytokine signaling activity measured as STAT3 activation. Importantly, conditioned medium from cells with double knockdown of VPS37A and VPS37B induced a strong activation of STAT3 as measured by Tyr705 phosphorylation in previously untreated cells (Fig. 5C). STAT3 (Tyr705) phosphorylation remained much less pronounced with conditioned medium from cells with single knockdown of VPS37A or VPS37B when compared to control cells (Fig. 5C). These results were reflected in the translocation of active STAT3 into the nucleus of conditioned medium exposed cells (Fig. S6B). We conclude that stalled simaphagy causes upregulation of both intra- and intercellular signaling events.

### Co-depletion of VPS37A and VPS37B induces dysfunctional endosomes and stalled simaphagy

**Figure 6.**
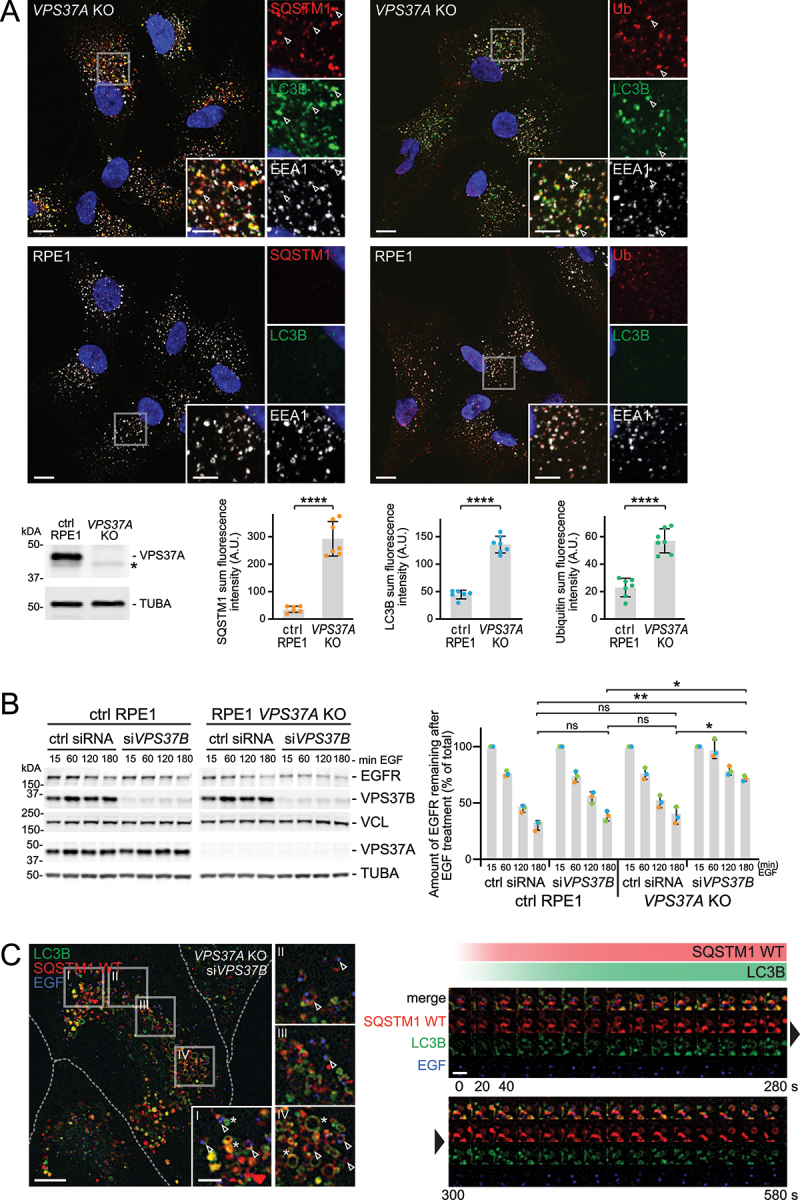
Loss of ESCRT-I subunits VPS37A and VPS37B leads to stalled simaphagy. (a) Representative immunofluorescence images of RPE-1 control or *VPS37A* KO cells. Loss of VPS37A leads to an increase in SQSTM1- and LC3B-positive objects and to an accumulation of ubiquitin (Ub) on endosomes. Below: western blot detecting full length VPS37A in RPE-1 control and *VPS37A* KO cells (asterisk indicates an unspecific background band detected by the VPS37A antibody). Quantification of a representative immunofluorescence experiment: sum fluorescence intensity of SQSTM1, LC3B and Ub objects show a significant increase in *VPS37A* KO cells. Mean ± SD of 7 images per condition and a total of 70-80 cells per condition. Two-tailed Student’s t test ****p<0.0001. Scale bar: 10 µm; 5 µm for insets. (B) Quantitative western blot analysis of EGFR degradation, after EGF stimulation in RPE-1 control cells, *VPS37A* KO cells and upon VPS37B knockdown. Left: representative western blot is shown. Degradation of EGFR is impaired upon *VPS37A* KO in combination with VPS37B knockdown. Right: western blot quantification showing residual EGFR after 15, 60, 120 or 180 min EGF stimulation. Values are displayed as percentage and normalized to the loading control and t = 15 min is set to 100%. Mean ± SD of three independent experiments. One-way ANOVA of 180 min timepoint *p < 0.1, **p < 0.01, ns = not statistically significant. (C) Movie stills of live-cell imaging experiments in *VPS37A* KO cells with additional knockdown of VPS37B. *VPS37A* KO cells stably expressing SNAP-LC3B and mCherry-SQSTM1 WT, were subjected to a 2 min pulse of EGF-Alexa647 [66] before imaging. SNAP-LC3B was visualized by incubation with SNAP-OregonGreen ligand prior to imaging. Cells display simaphagy events (arrowheads), as well as an accumulation of phagophores not containing endosomes (asterisk). Right: Timeline of a representative simaphagy event shows recruitment of mCherry-SQSTM1 WT and SNAP-LC3B. Scale bar: 10 µm; 3 µm for insets I-IV; 2 µm for timeline.

To reduce knockdown variability and potential off-target effects, we used CRISPR/Cas9 to generate a knockout (KO) of VPS37A in RPE-1 cells. *VPS37A* KO led to an increase in SQSTM1 and LC3B-positive objects, underlining the role of VPS37A in autophagy (Fig. 6A). This increase in SQSTM1 positive objects was only observed upon VPS37A loss but not by knockdown of other VPS37 isoforms (Fig. S7A). Further, we detected elevated ubiquitin staining, partially co-occurring with EEA1 signals in *VPS37A* KO cells, pointing towards an accumulation of ubiquitinated receptors on endosomes (Fig. 6A).

We quantified the amount of ubiquitinated receptors on endosomes by fluorescence microscopy. *VPS37A* KO cells accumulated on average 3 times more ubiquitinated receptors compared to control cells. The ubiquitin levels were further elevated in cells with *VPS37A* KO and VPS37B knockdown. Similarly, HGS[1-770]-positive endosomes displayed elevated ubiquitin levels on endosomes compared to wild-type HGS endosomes. These findings indicate that a pool of endosomes with a high quantity of ubiquitinated receptors on the limiting membrane are targets for autophagy (Fig. S7B, C).

In addition, we performed quantitative analyses of EGFR degradation and compared *VPS37A* KO and RPE-1 control cells, with and without additional knockdown of VPS37B. In general, EGFR degradation was slower in RPE-1 cells compared to HeLa cells. We observed a significant delay in EGFR degradation only in cells with *VPS37A* KO and knockdown of VPS37B. Depletion of VPS37A or VPS37B alone did not significantly delay degradation (Fig. 6B).

We also investigated the localization of PAG10-labelled EGFR in VPS37A and VPS37B co-depleted cells using electron tomography after 60 min of EGF stimulation. In approximately 67% of the endosomes the EGFR was still completely or partially scattered adjacent to the endosome limiting membrane. The PAG10 was found in aggregated clumps (similar as shown in Fig. 1C, left panel) inside (late) endosomes in only 29% of the cases (Fig. S7D).

To exclude that VPS37A and VPS37B depletion led to endosome rupture and subsequent autophagy of damaged endosomes, we stained for LGALS3 and LGALS8. As for HGS depletion (Fig. S2C, S2D), no LGALS3 or LGALS8 labelling was detected in VPS37A and/or VPS37B knockdown cells, indicating non-ruptured endosomes (Fig. S8A, S8B, S2C, S2D).

Next, we imaged and characterized simaphagy events in *VPS37A* KO cells. We used RPE-1 cells expressing SNAP-LC3B and mCherry-SQSTM1 WT at close to endogenous expression levels (Fig. S8C). We observed that *VPS37A* KO cells (with and without VPS37B knockdown) contained large numbers of small phagophores, which were not associated with EGF, therefore likely representing stalled autophagic structures unrelated to endosomes. Whether these phagophores were closed or open was not clear. Live cell imaging showed that these phagophores persisted over a period of > 15-20 min, frequently clustered, merged or deformed over time (Fig. S8D; Movie 8).

When focusing on EGF-associated SQSTM1- and LC3B-positive structures, i.e., potential stalled simaphagy events, we were able to detect those only upon *VPS37A* KO and additional knockdown of VPS37B (Fig. 6C; S8E; Movie 7). We imaged a total of 12 cells and detected 48 events, where mCherry-SQSTM1 WT and SNAP-LC3B were recruited to EGF-positive endosomes. We found between 1 and maximal 22 simaphagy attempts per cell during an imaging period of 30 min. The SQSTM1 and LC3B recruitment phase in *VPS37A* KO and VPS37B knockdown cells was similar as observed for HGS depleted endosomes. However, the growth phase was significantly longer. We found that the nascent phagophores frequently deformed, often displaying protruding LC3B-positive membranes, or failed to round up and close. Further, we observed that some of the simaphagy events turned out to be incomplete. In these cases, the endosome seemed to “escape” the open or deforming phagophores. Our live cell imaging further showed that these unclosed phagophores did not fuse with lysosomes (Fig. S8F). We conclude that only a loss of both VPS37A and VPS37B leads to occurrence of stalled simaphagy events due to their overlapping functions in endosomal sorting and the autophagosome-closing function of VPS37A.

### Increased directional cell migration in the absence of simaphagy

In our proteomics dataset we detected a large number of endosomal cargos, including numerous cell surface receptors that accumulated on dysfunctional endosomes. Depending on the receptor type this could result in sustained signaling that could either promote or inhibit cell migration. Therefore, we wondered if the capacity to perform simaphagy could have effects on cell migration.

**Figure 7.**
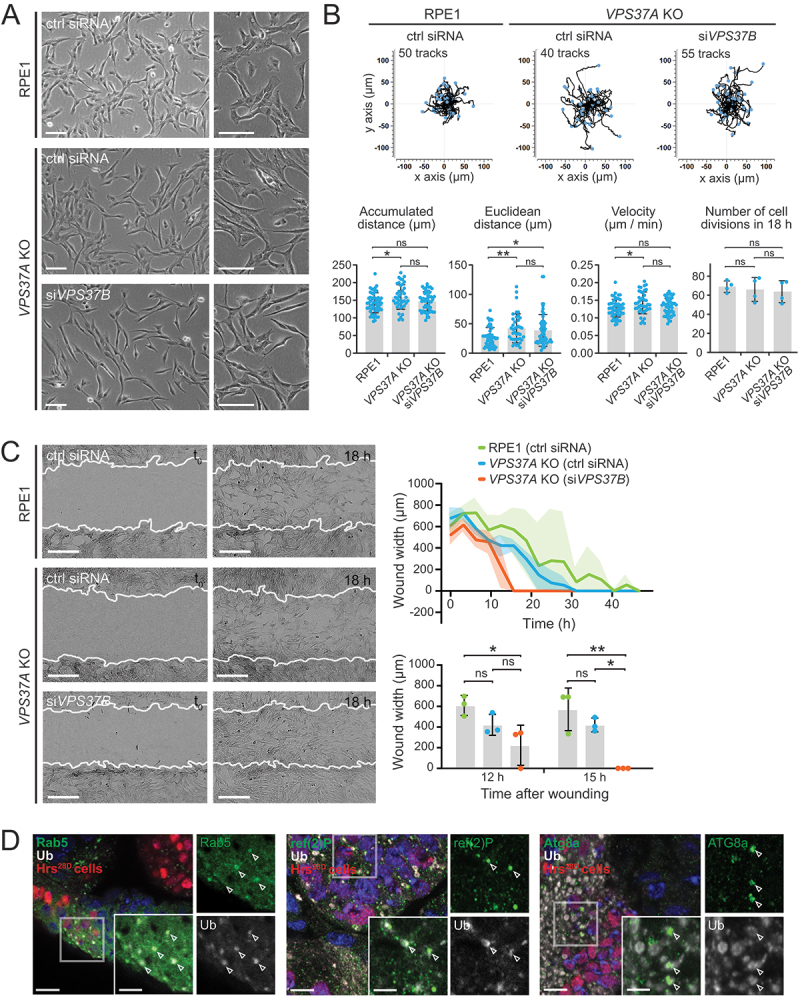
Simaphagy controls directed cell migration and can be detected *in vivo*. (a) Representative phase contrast images of RPE-1 control cells, *VPS37A* KO cells with and without knockdown of VPS37B using siRNA. Knockout of VPS37A leads to a more elongated cell morphology, a phenotype that is enhanced by knockdown of VPS37B. Scale bar: 100 µm. (b) Quantification of random migration patterns in RPE-1 control and *VPS37A* KO cells with and without VPS37B knockdown. 40-55 cells have been tracked for each condition over an imaging period of 18 h, with 20 min imaging intervals. Velocity, accumulative- and Euclidean distance are shown for each condition. The proliferation rate was manually scored for the imaging time of 18 h. One-way ANOVA *p < 0.05, **p <0.001, ns = not statistically significant. (c) Representative images and quantifications of wound healing assays performed with RPE-1 control and *VPS37A* KO cells with and without knockdown of VPS37B. *VPS37A* KO cells have an elevated migratory potential. This is further increased by a knockdown of VPS37B. Wound is shown at 1 h and 18 h after wounding (white line indicating cell front at t_0_ = 1 h). Scale bar: 300 µm. Average wound width over time and quantification of wound width at 12 h and 15 h are shown. Quantifications from 3 areas per condition. One-way ANOVA *p < 0.05, **p <0.001, ns = not statistically significant. (d) Representative immunofluorescence staining in Drosophila eye-antennal discs. Left: Clusters of Hrs^28D^ cells (marked by nuclear RFP) display enlarged Rab5-positive endosomes with accumulating ubiquitin. The ref(2)P (middle panel) and Atg8a (right panel) proteins are recruited to ubiquitin-labelled enlarged endosomes in HRS^28D^ larvae. Scale bar: 10 µm, insets 5 µm.

It has been reported that *VPS37A* KO leads to changes in cell morphology, promoting a mesenchymal like phenotype in SKOV3 and MDA-MB-468 cells [16]. In agreement with this, we observed that also RPE-1 *VPS37A* KO cells were slightly larger and more elongated compared to control RPE-1 cells. Surprisingly, with an additional knockdown of VPS37B the cells appeared even more spindle-like (Fig. 7A). We found only a marginal increase in random cell migration of *VPS37A* KO cells with or without additional VPS37B knockdown compared to parental RPE-1 cells, when measuring the average Euclidean distance (distance between start and end point), accumulated distance (total migrated distance) or velocity.

Having observed that random cell migration was largely unaffected under conditions of stalled simaphagy, we next measured directed cell migration with wound healing assays. It took control cells on average 36-42 h to close the 700-800 µm wide wound whereas *VPS37A* KO cells closed the wound slightly faster, between 27 to 30 h. Interestingly, in *VPS37A* KO cells with a VPS37B knockdown, i.e., under conditions of stalled simaphagy, only 12-15 h were required for wound closure (Fig. 7C). Since we did not observe significant differences in proliferation rates upon VPS37A and VPS37B depletion (Fig. 7B, Table 1 and 3), we assume that an increase in directed cell migration is responsible for the fast wound closure in VPS37A- and VPS37B-depleted cells. These experiments indicate that prolonged receptor signaling from endosomes has the potential to increase directed cell migration, and that this is counteracted by simaphagy.

**Table 1.** EM quantification.

	EGFR belowlimiting membrane	EGFR in clumps (MVB/lysosome)	EGFR in ILV	EGFR in small vesicle
RPE1 *VPS37A KO*, si*VPS37B* (250-nm section)	88.04	11.96	28.26	8.70
HeLa K HGS[1-770], WT SQSTM1 (250-nm section)	66.67	28.57	4.76	8.70

**Table 2.** Live cell imaging quantification.

	RPE1 GFP-LC3, mCH-SQSTM1 WT		RPE1 GFP-LC3, mCH-SQSTM1^M404V^		RPE1 GFP-LC3, mCH-SQSTM1ΔLIR		RPE1 GFP-LC3, mCH-SQSTM1ΔLIR		RPE1 *VPS37A* KO SNAP-LC3, mCH-SQSTM1 WT	
	ctrl siRNA	si*HGS*, si*SQSTM1*	ctrl siRNA	si*HGS*, si*SQSTM1*	ctrl siRNA	si*HGS*, si*SQSTM1*	ctrl siRNA	si*HGS*, si*SQSTM1*, si*NBR1*	ctrl siRNA	si*VPS37B*
imaged cells	12	26	3	8	6	8	3	4	10	36
tracked cells	7	20	2	7	3	8	1	3	10	12
endosomes tracked	14	96	11	81	11	80	15	23	15	51
SQSTM1 recruited	2	75	0	**24**	4	72	0	23	0	49
LC3 recruited	2	74	0	**25**	0	26	0	6	0	48
lysosomes docking/fusion	0	43	0	13	0	12	0	0	0	0
complete simaphagy events	0	36	0	6	0	10	0	3	-	not clear
average duration (simaphagy start to closure)	-	8.2 min	-	8.5 min	-	9.5 min	-	14.4 min	-	not clear
Standard deviation	-	3.2 min	-	4.4 min	-	4.9 min	-	9.6 min	-	not clear

bold numbers = very weak mCH-SQSTM1^M404V^ and GFP-LC3 signal (recruitment, most likely due to low knockdown efficiency of endogenous SQSTM1)

not clear = phagophores, frequently cluster and make it difficult to judge on closure; or the phagophores deform and open up again, with SNAP-LC3-positive membrane protruding into the cytosol.

**Table 3.** Proliferation quantification.

Number of cell divisions in 18 h (20-min imaging intervals)					Number of cells per imaging frame (at movie start t_0_)		
	RPE1	*VPS37A* KO			RPE1	*VPS37A* KO	
movie NR.	ctrl	ctrl	si*VPS37B*	movie NR.	ctrl	ctrl	si*VPS37B*
1	62	78	57	1	48	62	62
2	71	52	73	2	50	45	78
3	76	59	74	3	59	58	68
4	67	75	51	4	37	66	59
average	69.0	66.0	63.8	average	48.5	57.8	66.8
stdev	5.9	12.5	11.5	stdev	9.0	9.1	8.4

### Detection of simaphagy in vivo

Last, we addressed whether simaphagy can be detected *in vivo* by analyzing *Drosophila melanogaster* eye-antennal discs. Tissue specific mitotic recombination was utilized to induce clones of cells homozygous for the truncated allele *Hrs^28D^*, lacking ubiquitin binding and endosomal sorting ability. *Hrs^28D^* contains only the amino terminal third of the HGS protein, behaves genetically as a null mutation and has been shown to lead to enlarged endosomes and increased EGFR phosphorylation [45]. In *Hrs^28D^* cells (marked by nuclear RFP) we detected a pronounced ubiquitin signal on enlarged Rab5-positive endosomes, indicating receptor accumulation on these endosomes (Fig. 7D).

Upon *Hrs^28D^* mutation we further observed ref(2)P and Atg8a, the *D. melanogaster* homologs of mammalian SQSTM1 and LC3B, respectively, localizing to these ubiquitin-positive endosomes (Fig. 7D). The close proximity of ref(2)P and Atg8a to ubiquitin-positive endosomes therefore indicates simaphagy events, where autophagy targets ESCRT-dysfunctional endosomes. These experiments thus represent a first indication for simaphagy *in vivo*.

## Discussion

**Figure 8.**
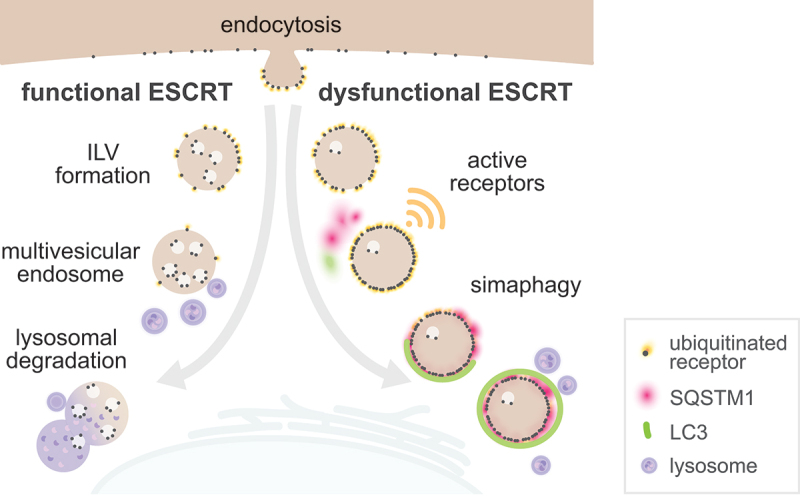
Model for simaphagy. Activated cell surface receptors are endocytosed and fed into the endosomal system. A fully functional ESCRT machinery mediates ILV formation and multivesicular endosome biogenesis, ultimately leading to lysosomal receptor degradation. A dysfunctional ESCRT machinery leads to accumulation of ubiquitinated receptors on the endosomal surface and prolonged receptor activation. Dysfunctional endosomes are recognized by the autophagy receptors NBR1 and SQSTM1, engulfed by LC3B-containing autophagic double membranes and degraded.

Here we describe for the first time that intact but hypersignaling endosomes are targeted by an autophagic process, simaphagy. These dysfunctional endosomes are generated by mutation or loss of essential ESCRT-0 and ESCRT-I subunits and accumulate ubiquitinated cargoes including surface receptors that can be active and signaling from the limiting endosome membrane. We found that ubiquitination of endosomal cargos is the recruiting signal for the autophagy machinery via the autophagy receptors SQSTM1 and NBR1. Dysfunctional endosomes are engulfed by a growing phagophore and thereby isolated from the cytosol. This simaphagy process is predominantly found in the perinuclear region (Fig. 8).

HGS is an endosome specific ESCRT subunit, and its manipulation should therefore only impact the endosomal system and avoid other ESCRT-mediated cellular processes. The striking enrichment of SQSTM1 and NBR1 in the proximity biotinylation approach with HGS[1-770] expressing cells was the first indication that autophagy could be involved in clearing ESCRT dysfunctional endosomes. SQSTM1 and NBR1 can both function as bridge between ubiquitinated cargo and the core autophagy machinery, enabling efficient recognition and sequestration within an autophagosome [36,37,46-50]. Further, we found that SQSTM1 and NBR1 can partially compensate for each other during simaphagy. SQSTM1 is recruited together with LC3B-positive phagophore membranes, which grow around and engulf dysfunctional endosomes. This process lasts between 7-13 min, until the LC3B signal decreases and the first lysosomes start to fuse with the resulting autophagosome. These simaphagy events are triggered by the autophagy initiators ULK1 and RB1CC1. Interactions between RB1CC1 and endosomal proteins such as RABEP1 might further facilitate autophagy initiation [24]. Because of the presence of ULK1 and RB1CC1, and the electron microscopic observation of double membranes around dysfunctional endosomes, we can conclude that simaphagy is distinct from conjugation of Atg8 proteins to single membranes (CASM) [51,52].

In imaging experiments, we observed SQSTM1 in proximity to dysfunctional endosomes, and electron microscopy showed that the SQSTM1 signal coincided with an accumulation of small vesicles. These vesicles might contribute to the growing phagophore. However, we also observed simaphagy events with hook-like protruding phagophores. We speculate that these are potentially connected to the ER, providing additional phagophore membrane during the growth phase. An interplay between ATG proteins, RAB GTPases, SNAREs and ESCRT proteins mediates the closure of the nascent autophagosome which can ultimately fuse with lysosomes for degradation of sequestered material [13,32,53,54].

Multiple pathways for selective removal of organelles have been investigated so far, including ER-phagy, lysophagy, lipophagy, mitophagy, nucleophagy, pexophagy, proteophagy and ribophagy [37,50,55]. These types of selective autophagy are frequently induced by ruptured organelles that initiate stress signals for the cell. Selective autophagy of ruptured endosomes, termed endosomophagy, has also been described [21-26].

Endosomophagy, like lysophagy, is activated by recognition of lumenal glycan groups by cytosolic galectins, which in turn recruit autophagy receptors [27-29]. An involvement of the endosomal RAB5-binding protein RABEP1 has also been reported [24]. We now observed that loss of essential ESCRT subunits triggers autophagy of endosomes without endosomal rupture and without involvement of galectins. Instead, autophagy of the resulting hypersignaling endosomes is triggered by accumulation of ubiquitinated cargoes on the endosome surface. Therefore, simaphagy represents a specialized form of endosomophagy and a safeguard mechanism to survey and clear a pool of aberrant endosomes with hyperaccumulation of active receptors.

VPS37 isoforms are particularly interesting in the context of simaphagy because their downregulation is frequently associated with cancers [15]. Only VPS37A is required for phagophore closure, but VPS37A and VPS37B have redundant functions in endosomal protein sorting [13, 43, 44, this paper]. This explains why stalled simaphagy events were only detected upon co-depletion of both VPS37A and VPS37B. We found that VPS37 loss contributes to strongly increased intercellular cytokine signaling (Fig. 5C) and directed cell migration (Fig. 7C). This suggests that simaphagy could have a tumor suppressor function, and it will be interesting to study this further.

## Materials and Methods

### Cell culture and generation of stable cell lines

HeLa (Kyoto) cells (RRID:CVCL_1922; kindly gifted by D. Gerlich) were grown according to ATCC guidelines in Dulbecco’s modified Eagle’s medium (DMEM) high glucose (Sigma, D0819-500ML or Gibco, 61965-026) supplemented with 10% fetal calf serum, 5 U/ml penicillin and 50 µg/ml streptomycin. Human retinal pigment epithelial (hTERT-RPE-1) cells (ATCC, CRL-4000) were grown in DMEM/F12 medium (Gibco, 31331-028) with 10% fetal bovine serum (FBS) (Sigma, F7524), 5 U/ml penicillin and 50 µg/ml streptomycin. HeLa (Kyoto) and hTERT-RPE-1 cells were maintained at 37°C, under 5% CO_2_, characterized by genotyping and regularly tested for mycoplasma contamination. Stable HeLa (Kyoto) expressing GFP-HGS WT, GFP-HGS[1-770] were previously described in [5] and stable hTERT-RPE-1 expressing GFP-LC3B were described in [32].

All other stable cell lines were lentivirus-generated pools, by using plasmids coding for pCDH-EF1alpha-IRES-BLAST-APEX2-GFP-HGS WT, pCDH-EF1alpha-IRES-BLAST-APEX2-GFP-HGS[1-770], pCDH-PGK-IRES-NEO-mCherry-SQSTM1 WT, pCDH-PGK-IRES-NEO-mCherry-SQSTM1^M4^°^4^, pCDH-PGK-IRES-NEO-mCherry-SQSTM1ΔLIR, pCDH-PGK-IRES-NEO-SNAP-LC3B, pCDH-PGK-IRES-BLAST-mCherry-HGS WT and pCDH-PGK-IRES-BLAST-mCherry-HGS[1-770]. The weak *PGK* promoter was used for transgene expression in order to achieve low expression levels. Third-generation lentivirus was generated as previously published in [56]. Briefly, mCherry or eGFP fusions were generated as Gateway Entry plasmids using standard molecular biology techniques. From these vectors, lentiviral transfer vectors were generated by recombination into pCDH Destination vectors using a Gateway LR reaction. VSV-G-pseudotyped lentiviral particles were packaged using a third-generation packaging system [57]. Cells were then transduced with low virus titers, and stable expressing populations were generated by antibiotic selection. Some of the stable cell lines were sorted by flow cytometry to obtain pools of cells with close to endogenous levels of expression.

In this study we used the following stable cell lines: HeLa GFP-HGS WT; HeLa GFP-HGS[1-770]; HeLa APEX2-GFP-HGS WT; HeLa APEX2-GFP-HGS[1-770]; RPE-1 eGFP-LC3B, mCherry-SQSTM1 WT; RPE-1 eGFP-LC3B, mCherry-SQSTM1^M4^°^4V^; RPE-1 eGFP-LC3B, mCherry-SQSTM1ΔLIR; RPE-1 *VPS37A* KO, SNAP-LC3B, mCherry-SQSTM1 WT; RPE-1 mCherry-HGS WT; RPE-1 mCherry-HGS[1-770].

### *Generation of* VPS37A *KO cell lines*

The guide (g)RNA sequence 5′- CACCGAGGAGGCGCTCTTGGTCAG -3′ was used to generate a ribonucleoprotein (RNP) complex, following the manufacturers protocol (all reagents and protocols were purchased from IDT, https://eu.idtdna.com). The VPS37-RNP complex was nucleofected into hTERT-RPE-1 cells and sorted by flow cytometry into single cells and further grown in 24-well plates. The resulting colonies were assayed by western blotting and sequencing of cloned PCR fragments from a genomic PCR flanking the predicted Cas9 cleavage site. The PCR primers for the genomic PCR were 5′- CTCGGGGAGCGCAGGCAGGACAGGC -3′ and 5′- CAGTCAGCAGCTATGTGTCAGGAGG -3′, with a product length of 551bp, which was cloned into pJet vector (Thermo Fisher Scientific, K1232) for Sanger sequencing. Three clones that lacked the expression of VPS37A were identified by western blotting. Genomic PCR followed by Sanger sequencing showed that one cell line had two genomic alterations. One of the two alleles had a deletion (adenine, cytosine), whereas the other allele had an insertion (adenine), both mutations resulting in a frameshift. Sequencing of the other cell lines showed that, in one cell line we found both alleles with an insertion (adenine) and in the other cell line we found both alleles with a deletion (adenine, cytosine). The cell line containing both, insertion and deletion was further validated by immuno-florescence microscopy and chosen for subsequent experiments.

### APEX2-mediated proximity biotinylation

HeLa (Kyoto) cells stably expressing APEX2-eGFP-HGS WT or APEX2-eGFP-HGS[1-770] were grown in 10-cm dishes and subjected to a 5-day knockdown using siRNA targeting endogenous HGS. On day 3 in the knockdown treatment, cells were trypsinized and seeded in 15-cm dishes and further grown to 80% confluency until day 5. The cells were incubated at 37°C in 500 µM Biotin-Phenol (Iris Biotech GmbH, LS-3500) for 2 h and kept unstimulated or stimulated for 15 min with EGF (final concentration 50 ng/µl; Sigma, E9644). Cells were then incubated with room temperature warm DMEM-H_2_O_2_ (final concentration 2 mM H_2_O_2_ [Merck, H1009]) for 2 min and then washed 4 x with ice cold quencher solution (10 mM sodium ascorbate [Merck, PHR1279], 5 mM trolox [Merck, 238813], 10 mM sodium azide [VWR, AA14314-36] in PBS [Merck, D8537]). During the last wash the cells were left in the quencher solution at 4°C for 20 min. Cells were lysed for 10 min with ice cold RIPA buffer (50 mM TRIS, 150 mM NaCl, 0.1% SDS, 0.5% NaDOC [Merck, D6750], pH 7.5) supplemented with protease inhibitors (Roche, 05056489001), 1 mM PMSF [Merck, P7626], 10 mM sodium azide, 10 mM sodium ascorbate and 5 mM trolox). The lysates were collected and centrifuged at 20,000 g at 4°C for 15 min to pellet the DNA. The supernatant was transferred to desalting columns (Zeba^TM^; Thermo Fisher Scientific, 89890, 89893) to eliminate the free biotin. The lysates were incubated with Streptavidin Dynabeads (Invitrogen, M-280) for 2 h at 4°C. The beads were washed carefully on ice: twice with RIPA buffer, one wash with 1 M KCl, one wash with 0.1 M Na_2_CO_3_, followed by two washes with 2 M urea (Merck, U5378) in 10 mM Tris-HCl (pH 8.0, freshly prepared), one wash with RIPA buffer and five washes with ice-cold PBS. The samples were kept at 4°C before being further processed for mass spectrometry analysis.

### LC–MS/MS, protein identification and label-free quantification

Beads containing bound proteins were resuspended in 50 mM ammonium bicarbonate buffer and 0.04% ProteaseMax surfactant (Promega, V2072), reduced with 5 mM DTT for 1 h at 56°C followed by alkylation with 15 mM iodoacetamide in final volume of 100 µl for 1 h at room temperature. The samples were digested overnight with sequencing-grade trypsin (Promega, V5111) at 37°C, using 1 µg trypsin. Reaction was quenched by adding 1% trifluoracetic acid to the mixture. Peptides were cleaned for mass spectrometry using the STAGE-TIP method and a C18 resin disk (3 M Empore, 55004-098) [58]. The samples were analyzed in technical triplicates on an Easy nLC1000 nano-LC chromatography (LC) system connected to a quadrupole-Orbitrap QExactive Plus mass spectrometer (ThermoElectron, Bremen, Germany) equipped with a nano-electrospray ion source (EasySpray/Thermo). For liquid chromatography separation we used an EasySpray column (C18, 2 µm beads, 100 Å, 75 μm inner diameter) (Thermo) capillary of 25 cm bed length. (The flow rate used was 0.3 μl/min, and the solvent gradient was 2–7% solvent B in 5 min, then to 30% solvent B in 60 min. Solvent A was aqueous 1% formic acid, whereas solvent B was 100% acetonitrile in 0.1% formic acid. Column temperature was kept at 60°C.

The mass spectrometer was operated in the data-dependent mode to automatically switch between mass spectrometry (MS) and MS/MS acquisition. Survey full scan MS spectra (from m/z 300–1750) were acquired in the Orbitrap with a resolution (R)=70,000 at m/z 200, after accumulation to a target of 3,000,000 ionsper quadruple. The method used allowed sequential isolation of the most-intense multiple-charged ions – up to ten, depending on signal intensity – for fragmentation on the higher energy C-trap dissociation (HCD) cell using high-energy collision dissociation at a target value of 100,000 charges or maximum acquisition time of 100 ms. MS/MS scans were collected at 17,500 resolution at the Orbitrap cell. Target ions already selected for MS/MS were dynamically excluded for 305 s. General MS conditions were: electrospray voltage, 2.1 kV; no sheath and auxiliary gas flow, heated capillary temperature of 250° C, normalized HCD collision energy 25%.

MS raw files were submitted to MaxQuant software version 1.6.1.0 for protein identification and label free quantification [59]. Parameters were set as follows: protein N-acetylation and methionine oxidation as variable modifications. First search error window of 20 ppm and mains search error of 6 ppm. Trypsin without proline restriction enzyme option was used, with two allowed miscleavages. Minimal unique peptides were set to 1, and false-discovery rate (FDR) allowed was 0.01 (1%) for peptide and protein identification. Label-free quantification was set with a retention time alignment window of 3 min. The Uniprot human database was used (downloaded September 2018). Generation of reversed sequences was selected to assign FDR rates. Filtering of the data and student t-test was performed in the software Perseus (version 1.6.6.0 and 1.6.1.3). Data was transformed and at least 25% valid values were required for each sample group. Missing values were imputed from normal distribution. Student t-test with permutation-based FDR cutoff p< 0.05 was used.

### Knockdown using siRNA

Cells were transfected using Lipofectamine RNAiMAX transfection reagent (Life Technologies, 13778-150) following the manufacturer’s instructions. siRNAs were purchased from Ambion® (Thermo Fisher Scientific) and contained the Silencer Select modification. Non-targeting control Silencer Select siRNA (predesigned, 4390844) was used as control. Cells were transfected with 50 nM siRNA targeting human *HGS* (5′-GCACGUCUUUCCAGAAUUC-3′) for 5 days.

siRNAs from Dharmacon ON-TARGET plus were used to target *SQSTM1* (5′- GCAUUGAAGUUGAUAUCGAUUU -3′), *RB1CC1* (5′- GGAGUGGGCUGGUGCUUUA -3′), ULK1 (5′- UCACUGACCUGCUCCUUAA -3′) and *NBR1* (5′- GAACGUAUACUUCCCAUUG -3′). Cells were transfected 48-72 h, using 25 nM final concentration for each siRNA respectively. VPS37B was targeted by using 100 nM final concentration of Dharmacon ON-TARGET plus *VPS37B* (5′- AGUUGUGUGUGCCGGGUUA -3′), for 3 – 5 days. Dharmacon ON-TARGETplus Non-targeting siRNA was used as control (predesigned, catalogue number D-001810-01).

*VPS37A* was targeted with a 5-day knockdown, using a Dharmacon ON-TARGET plus pool siRNA (L-016816-01-0005), with a final concentration of 50 nM. Dharmacon ON-TARGET plus Non-targeting Pool (D-001810-10-20) was used as control siRNA.

The *HGS* transgenes in the stable cell lines are mouse sequences and the *SQSTM1* transgenes contain two silent point mutation (WT siRNA target sequence: 5′- GCATTGAAGTTGATATCGAT -3′; siRNA resistant sequence: 5′- GCATTGAGGTAGATATCGAT -3′) making them resistant towards the siRNAs.

### Immunostaining and antibodies

If not stated otherwise, cells were grown on coverslips and were permeabilized with 0.05% saponin (Merck, S7900) in PEM buffer (80 mM K-PIPES, pH 6.8, 5 mM EGTA, and 1 mM MgCl_2_) for 5–10 min on ice to decrease the fluorescent signal from the cytosolic pool of proteins before fixation in 4% EM-grade paraformaldehyde for 15 min [60]. Cells were washed twice in PBS and once in PBS containing 0.05% saponin before staining with the indicated primary antibodies for 1 h at room temperature. After washing three times in 0.05% saponin in PBS, cells were stained with secondary antibodies for 1 h in the dark at room temperature and washed three times in PBS. The cells were mounted in Mowiol (Merck, 81381) containing 2 mg/ml Hoechst 33342 (Sigma, H3570). Immunostaining of STAT3 was performed after HeLa cells were incubated with conditioned medium for 15 min. The cells were fixed with 4% EM-grade paraformaldehyde (Polyscience Inc., 18814) for 15 min at room temperature. Cells were washed three times in 1x PBS and once with 1x PBS containing 0.5% Triton X-100 (Sigma, T9284) before incubating with the STAT3 primary antibody overnight at 4°C. After washing three times in 0.5% Triton X-100 in PBS cells were stained with secondary antibodies as described above.

Antibodies used in this study: mouse anti-GFP (clones 7.1 and 13.1, 11814460001, IF 1:400), mouse anti-TUBA/alpha-tubulin (T5168; WB 1:20000) and mouse anti-VCL/vinculin (Clone HVIN-1, WB 1:3000; Merck, V9131) were from Sigma-Aldrich. Human anti-EEA1 serum, was a gift from Ban-Hock Toh, Melbourne, Australia, rabbit anti-HGS/HRS (IF 1:50-100, WB 1:1000) has been described previously [18]. Sheep anti-EGFR (20-ES04; IF 1:4000, WB 1:7000) was from Fitzgerald, mouse anti-EGFR (555996; extracellular labelling of EGFR) was from Pharmingen, goat-anti-mCherry (AB0040-200; IF 1:400, WB 1:1000) from Acris antibodies. Human LGALS3/Galectin-3 Alexa Fluor 488-conjugated antibody (R&D Systems, IC1154G). Goat LGALS8/Galectin-8 (R&D Systems, AF1305; IF 1:200). SQSTM1 was detected using rabbit anti-SQSTM1 (IF 1:500; WB 1:1000; MBL, PM045), or guinea pig anti-SQSTM1 (IF 1:100, WB 1:1000; Progen, GSQSTM1-C). rabbit anti-LC3B (IF 1:500, WB 1:1000) was from MBL (PM036). Mouse anti-ubiquitin (clone FK2, IF 1:400) was from EMD Merck Millipore (04-263). mouse anti-NBR1 (clone 4BR, IF 1:100) was from Santa Cruz Technology (sc-130380), mouse anti-NBR1 (6B11, WB 1:500 – 1:1000) was from Abnova (H00004077-M01). Rabbit anti-RB1CC1/FIP200 (IF 1:50, WB 1:1000) was from Proteintech (17250-1-AP) and rabbit anti-ULK1 (D8H5; IF 1:200) from Cell Signaling Technology (8054). Rabbit anti-VPS37A (11870-1-AP; IF 1:100, western blot 1:1000-2000) and rabbit anti-VPS37B (15653-1-AP-20; WB 1:1000-2000) were from Proteintech. Rabbit anti-phospho-EGFR (Tyr845) (2231; WB 1:1000), rabbit anti-AKT (9272; WB 1:1000), rabbit anti-phospho-AKT (Ser437) (4058; WB 1:1000), rabbit anti-MAPK1/ERK2-MAPK3/ERK1 (9102; WB 1:2000) and rabbit anti-phospho-MAPK1/ERK2-MAPK3/ERK1 (Thr202/Tyr204) (9106; WB 1:2000), mouse anti-STAT3 (9139; WB 1:1000, IF 1:3000) and rabbit anti-phospho-STAT3 (Tyr705) (9145; WB 1:1000) were from Cell Signaling Technology. HRP-streptavidin (WB 1:5000) was from Jackson (016-030-084). All secondary antibodies used for immunofluorescence studies were obtained from Jacksons ImmunoResearch Laboratories (711-545-152, 709-605-149, 705-545-147, 715-545-151, 706-545-148, 706-605-148, 711- 605 -152, 43250) or from Molecular Probes (Life Technologies, A-21099, A21432). Secondary antibodies used for western blotting were obtained from LI-COR Biosciences GmbH (926-68072, 926-32212, 926-68073, 926-32213, 926-68074, 926-32411), and horseradish peroxidase-conjugated secondary antibodies were from Jackson (115-035-003, 111-035-144).

### Assay for endosome rupture with LGALS3 and LGALS8

HeLa cells were subjected to knockdown using siRNA as stated above and grown on coverslips for immunostaining. A lysosomotropic drug L‐leucyl‐L‐leucine methyl ester (Cayman Chemical; 16008) has been used to induce endosome/lysosome damage. LLOMe was dissolved in dimethyl sulfoxide (DMSO) (Merck, D2650) and stored at −20°C. Indicated cells have been incubated with 250 µM LLOMe in DMEM at 37°C for 15 min, followed by fixation using 4% EM-grade paraformaldehyde for 15 min at room temperature. Cells have been stained using human LGALS3 (Galectin 3) Alexa Fluor 488-conjugated or goat LGALS8 (Galectin 8), human anti-EEA1 and rabbit anti-HGS antibodies and immunostaining protocol as described above.

### Western blotting

Cells were washed twice with ice cold PBS and lysed with 2 x sample buffer (125 mM Tris-Cl, pH 6.8 [Sigma, T1378], 4% SDS [Sigma, L-5750], 20% glycerol [Sigma, G5516], 200 mM DTT [Merck, D0632], 0.004% bromophenol blue) supplemented with protease inhibitor cocktail (cOmplete, EDTA free; Roche, 05056489001) and for signaling experiments supplemented with phosphatase inhibitors (phosSTOP; Roche, 04906837001). Lysates were subjected to SDS-gel electrophoresis on 10% or 4-20% gradient gels (mini- or midi-PROTEAN TGX; Bio-Rad, 5671095, 5671035, 5671034, 5671094, 4561036, 4561095). Proteins were transferred to PVDF membranes (TransBlot® Turbo^TM^ LF PVDF; Bio-Rad, 170-4275, 170-4274) followed by blocking and antibody incubation in 2% BSA (Merck, BSAV-RO 10735094001) in Tris-buffered saline (pH 7.5), with 0.05% Tween-20 (Sigma, P1379). Membranes incubated with fluorescent secondary antibodies (LI-COR) were developed using an Odyssey infrared scanner (LI-COR), or incubated with horseradish peroxidase-conjugated antibodies and developed using a Clarity Western ECL substrate solution (Bio-Rad, 1705060) and ChemiDoc XRS+ imaging system (Bio-Rad). Quantification and analysis of immunoblots was performed with Odyssey Software (version 3.0.30) or ImageJ (version 1.52p).

### Immunogold labelling and electron microscopy

HeLa (Kyoto) and hTERT-RPE-1 cells were grown on poly-L-lysine-coated (Merck, P7890) and FN1 (fibronectin 1)-coated (Merck, F2006) sapphire discs, respectively, and subjected to EGFR-immunogold-labelling, high-pressure freezing and electron microscopy as described in [5]. In brief, cells were first washed with ice cold PBS and incubated on ice with an antibody recognizing the extracellular part of EGFR (mouse anti-EGFR; Pharmingem, 555996). This will label the newly internalized EGFR following EGF stimulation. After washing four times with ice cold PBS, the cells were incubated with protein A-conjugated 10-nm gold conjugate (UMC Utrecht Department of Cell Biology), binding to the mouse primary antibody. The cells were washed four times with ice cold PBS and stimulated with EGF (50 ng/ml) in warm DMEM for the indicated times before high-pressure freezing. Sapphire discs were high-pressure frozen using a Leica HPM100. Freeze substitution was performed as follows: sample carriers designed for sapphire discs (Leica 16701154 and 16701157) were filled with 4 ml of freeze substituent (0.1% [w:v] uranyl acetate in acetone, 1% H2O) and placed in a temperature-controlling AFS2 (Leica) equipped with an FPS robot. Freeze substitution was conducted at −90°C for 48 h before the temperature was raised to −45°C, over a time span of 9 h. The samples were kept in the freeze substituent at −45°C for 5 h before washing 3 times with acetone followed by a temperature increase (5°C per hour) to −35°C, and then infiltrated with increasing concentrations of Lowicryl HM20 (Electron Microscopy Science/EMS, 14340) 10%, 25%, 75%, 4 h each. During the last two steps, temperature was gradually raised to −25°C before infiltrating 3 times with 100% Lowicryl (10 h each). Subsequent ultraviolet polymerization was initiated for 48h at −25°C, and the temperature was then evenly raised to +20°C (5°C per hour). Polymerization then continued for another 24 h at 20°C. Serial sections with 250 nm thickness were cut on an Ultracut UCT ultramicrotome (Leica, Germany) and collected on formvar-coated slot grids. On-section labelling was performed with a SQSTM1-antibody raised in rabbit (MBL, PM045) and visualized with protein A-conjugated 5-nm gold conjugate (UMC Utrecht, PAG5NM). Electron tomograms were collected with a Thermo ScientificTM TalosTM F200C microscope and single-axis tilt series were taken between −60° and 60° tilt angles with 2° increment and recorded with a Ceta 16M camera. Tomograms were computed using weighted back projection using the IMOD package. Display and segmentation of tomograms were also performed using IMOD software version 4.11 [61].

### EGFR degradation assay

HeLa (Kyoto) cells or hTERT-RPE-1 cells were seeded and subjected to knockdown using siRNA for the indicated times. On the experiment day, cells were incubated for 1 h with cycloheximide (10 µg/ml; Sigma, C7698) in DMEM at 37°C. Cells were then stimulated with EGF (50 ng/ml; Sigma, E9644) for 15 min or 60 min in DMEM, while cycloheximide remained present during the pulse-chase experiment, to prevent *de novo*-synthesis of EGFR. The cells were either fixed in 4% paraformaldehyde and stained with antibodies for immunofluorescence and confocal microscopy or lysed and subjected to western blotting as described above.

### EGFR, AKT and MAPK/ERK phosphorylation experiments

HeLa (Kyoto) cells were seeded and subjected to knockdown using siRNA for the indicated times. Cells were maintained in DMEM supplemented with 10% FBS during the knockdown period and incubated in DMEM without FBS overnight (approx. 13-16 h). On the experiment day, cells were incubated for 1 h in warm DMEM without FBS containing cycloheximide (10 µg/ml) at 37°C. The cells were then pulsed with warm DMEM containing 5 ng/ml EGF for 5 min at 37°C, followed by a quick wash using warm PBS and a chase for the indicated times in DMEM supplemented with 10% FBS and cycloheximide (10 µg/ml) at 37°C. Cells were lysed with 2x sample buffer containing proteinase and phosphatase inhibitors and subjected to immunoblotting as described above.

### STAT3 phosphorylation experiments using conditioned medium

HeLa (Kyoto) cells were seeded and subjected to knockdown of relevant proteins using siRNA for the indicated times. Cells were maintained in DMEM supplemented with 10% FBS during the knockdown period. Cells were grown to 80% confluency, washed two times with warm PBS and one time with DMEM without FBS and incubated in DMEM without FBS for approx. 20-24 h before harvesting the conditioned medium. On the experiment day, the conditioned medium was carefully removed, centrifuged at 675 x g for 10 min to remove cellular components and filtered using a 0.2 µm pore size. The conditioned medium was applied to previously untreated HeLa cells for the indicated times (0, 15, 30 or 60 min) and incubated at 37°C. The cells were lysed with 2x sample buffer containing proteinase and phosphatase inhibitors and subjected to immunoblotting as described above.

### Fly genetics and histology

*D. melanogaster* lines and crosses were raised on standard potato mash fly food (32.7 g dried potato powder, 60 g sucrose, 27.3 g dry yeast, 7.3 g agar, 4.55 ml propionic acid, and 2 g nipagin per l) at 25^0^C and 70% humidity.

Loss of function clones of cells homozygous for *Hrs^28D^* [45] were generated in eye-antennal discs by the mosaic analysis with a repressible cell marker (MARCM) strategy [62] by crossing the following lines: *w^1118^/w^1118^; FRT40, tubP-Gal80/CyO; ey-Flp, Act>CD2>Gal4, UAS-His2A-mRFP/TM6* and *w^1118^/w^1118^; FRT40A, Hrs^D28^/Cyo; +/+* or *w^1118^/w^1118^; FRT40A Hrs^D28^/Cyo; UAS-Atg^GL^°°°^47^ RNAi/TM6* lines. All fly lines were obtained from the Bloomington Drosophila Stock Center (Bloomington, Indiana, US).

Eye-antennal discs were dissected out from late L3 staged (6 days after egg laying) larvae and fixed for 20 min in 4% paraformaldehyde-PBS at RT and washed for 3 x 5 min in PBT (0,5% Triton X-100 in PBS) at RT. Subsequently, samples were incubated in primary antibody solution (primary antibody in 5% FBS-PBT) overnight at 4°C. On the next morning, samples were washed for 3 x 5 min in PBT and incubated in secondary antibody solution (secondary antibody in 5% FBS-PBT) for 3 h at RT. Then, samples were washed 2 x 5 min in PBT and 2 x 5min in PBS at RT. The ready samples were mounted in Vectashield (VECTOR Laboratories, H-1000) and analyzed with Zeiss LSM 880 confocal microscope. The primary antibodies used were the following: mouse monoclonal anti-ubiquitin FK2 (1:400; Merck, 04-263), rabbit polyclonal anti-Rab5 (1:100; Abcam, ab31261), rabbit polyclonal anti-ref(2)P (1:300) [63] and rabbit monoclonal anti-Gabarap+GabarapL1+GabarapL2 (1:300; Abcam, ab109364) that also recognizes Drosophila Atg8a [64]. We used Alexa Fluor- conjugated secondary antibodies (Jacksons ImmunoResearch Laboratories, 715-545-151, 711-545-152; Molecular Probes, Life Technologies, A10042, A10037) specific for the appropriate species.

### Cell migration and proliferation experiments

Prior to imaging, hTERT-RPE-1 control cells and *VPS37A* KO cells have been subjected to knockdown using VPS37B or control siRNA. For wound healing assays, cells have been seeded 8h prior wounding in 96-well plates. We used 25.000 cells/well to reach 90-100% confluency. Wounds have been generated using the IncuCyte® WoundMaker™ following the manufacturer’s protocol and cells were imaged for 72 h with intervals of 3 h. For migration experiments, cells were treated with the indicated siRNAs and seeded in 6 wells (30,000 cells/well to achieve single cell distribution). Cell migration was monitored for 18 h with intervals of 20 min. To assess the proliferation rate the obtained images from single cell migration were analyzed by visual inspection and cell divisions were manually scored. Cells have been imaged using the IncuCyte® Zoom and analyzed with the IncuCyte® Zoom, Scratch Wound Cell Migration & Invasion Analysis Metrics (Essen Bio Science Inc.; version 2018A). Cell migration was manually tracked in ImageJ and analyzed and plotted using ibidi®: Chemotaxis and Migration Tool 2.0 (http://www.ibidi.de/applications/ap_chemo.html).

### Live cell imaging

hTERT-RPE-1 cells stably expressing GFP-LC3B-mCherry-SQSTM1 WT, or GFP-LC3B-mCherry-SQSTM1^M4^°^4V^, or GFP-LC3B-mCherry-SQSTM1ΔLIR have been subjected to knockdown of HGS, endogenous SQSTM1 and the corresponding controls using siRNA as described above. Similarly, hTERT-RPE-1 cells stably expressing SNAP-LC3B-mCherry-SQSTM1 WT, have been subjected to a VPS37B knockdown using the indicated siRNAs and controls. Cells were grown on MatTek 35 mm glass-bottom dishes (MatTek Corporation, P35G-1.5-20-C) in DMEM /F12 supplemented with 10% FBS. Cells expressing SNAP-LC3B-mCherry-SQSTM1 WT, were labeled 30 min prior to imaging with fluorescent SNAP ligand (SNAP-Cell Oregon Green; NEB, S9104S) according to the manufacturer’s protocol. Cells were stimulated for 2 min with 200 ng/ml EGF-Alexa Fluor 647 (Molecular Probes, Thermo Fisher, E-35351), washed 3 times with warm DMEM F12 (with 10% FBS). Cells were maintained at 37°C by a heated stage and objective, stable 5% CO_2_ and humidity were provided by a CO_2_ mixer (Okolab) during the whole imaging period. Live-cell imaging was performed on an OMX V4 system (DeltaVision OMX Microscope Applied Precision, GE Healthcare) equipped with an Olympus 60 × Plan Apochromat 1.42 numerical aperture objective, three cooled PCO.edge sCMOS cameras, a solid-state light source (InsightSSI) and a laser-based autofocus. Three color live-cell imaging was done in conventional mode and movies of 30 min length (5 s between frames) have been acquired. Hardware alignment is done twice a year by GE Healthcare service personal. The xyz alignments are controlled regularly and are adjusted if necessary by the staff of Advanced Light Microscopy Core Facility using bead slides. To guarantee optimal xy alignment we tested and adjusted if necessary before the imaging experiments by using the “GE Image Registration Slide”. The acquired images were deconvolved and aligned using the supplied Softworx software (GE healthcare) and further processed in ImageJ. If required movies have been background corrected and de-bleached with ImageJ. A custom-made python script was used to manually track individual EGF-positive endosomes in ImageJ and measure the fluorescence intensity over time (described in [5]. The image analysis scripts are available on https://github.com/koschink/Wenzel_et_al_2018.

### Confocal microscopy

Stained coverslips were examined with a Zeiss LSM 710, or 780 confocal microscope (Carl Zeiss) equipped with an Ar laser multiline (458/488/514 nm), a DPSS-561 10 (561 nm), a laser diode 405-30 CW (405 nm), and a HeNe laser (633 nm). The objective used was a Zeiss Plan-Apochromat 63 ×/1.40 Oil DIC M27 (Carl Zeiss). Image processing was performed with ImageJ software (version 1.52p). Intensity settings for the relevant channels were kept constant during imaging. Images shown in figures are representative of at least three independent experiments

### Statistical analysis and considerations

The number of individual experiments, the number of cells and the number of endosomes analyzed are indicated in the figure legends. We tested our datasets for normal distribution and chose an appropriate test accordingly using GraphPad Prism version 5.01. The unpaired, two-tailed Student’s *t*-test or one-way analysis of variance [65] followed by suitable post hoc tests were used to test for significant differences between samples. P-values are indicated for each experiment.

## Supplementary Material

Supplemental Material
