# Supplementary material for: Removal of hypersignaling endosomes by simaphagy
Source: Autophagy. 2023 Oct 16;20(4):769–91. doi: 10.1080/15548627.2023.2267958 (PMC11062362; doi:10.1080/15548627.2023.2267958)
Supplement: Supplemental Material [file KAUP_A_2267958_SM7544.zip › Supplementary_figures_revised R5.docx]

**
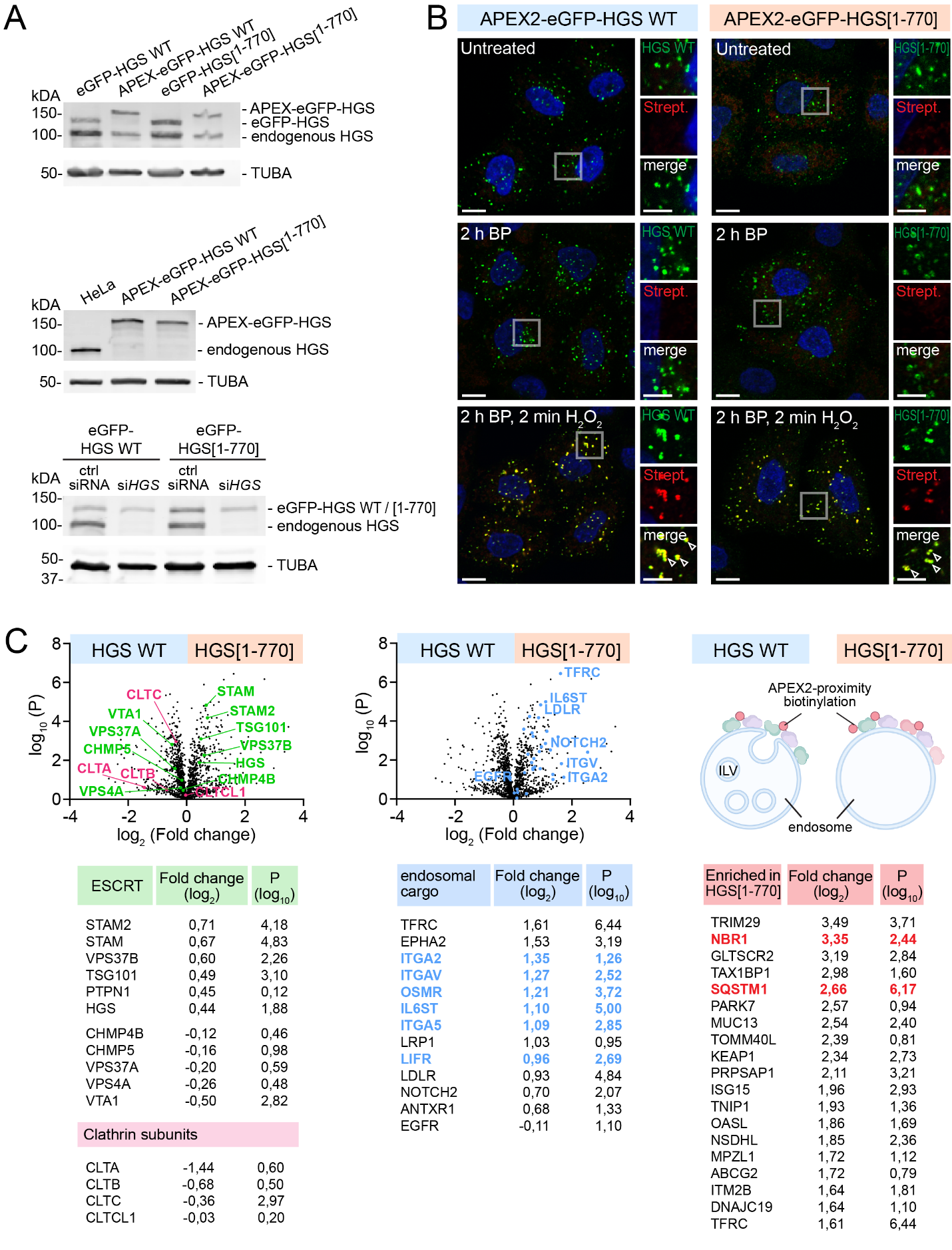
**

**Figure S1.** Autophagy receptors and endosomal cargo accumulate on HGS-mutant endosomes. (**A**) Western blot comparing the protein levels in cells expressing endogenous HGS and eGFP-HGS WT or -HGS[1-770] and in cells with stable expression of APEX2-eGFP-HGS WT or -HGS[1-770] (upper panel). Western blot with knockdown efficiency of endogenous HGS in APEX2-eGFP-HGS WT or -HGS[1-770] cells for proteomics experiments (middle panel) and in eGFP-HGS WT or -HGS[1-770] cells for immuno-staining in **Figure 1B** (lower panel). (**B**) Biotinylation test in cells stably expressing APEX2-eGFP-HGS WT or -HGS[1-770] and depleted for endogenous HGS. Immunofluorescence shows co-occurring HGS and streptavidin labelling in untreated cells, 2 h Biotin-Phenol (BP) treated cells and 2 h BP + 2min H_2_O_2_ treated cells. Scale bar: 10 µm; 5 µm for insets. (**C**) Left: Overview over enriched ESCRT- (green) and clathrin subunits (magenta). All clathrin subunits are enriched in the wild-type HGS sample, but absent in HGS[1-770]. Middle: Volcano plots showing the distribution of representative endosomal receptors and their enrichment in the HGS[1-770] sample (highlighted in blue). Right: Overview on the first 20 protein hits from APEX2-proximity biotinylation and mass spectrometry analysis, with the highest fold change (log_2_). NBR1 and SQSTM1 are found in the top five protein hits. Fold change (log_2_) and P-values (log_10_) for the representative proteins are stated.


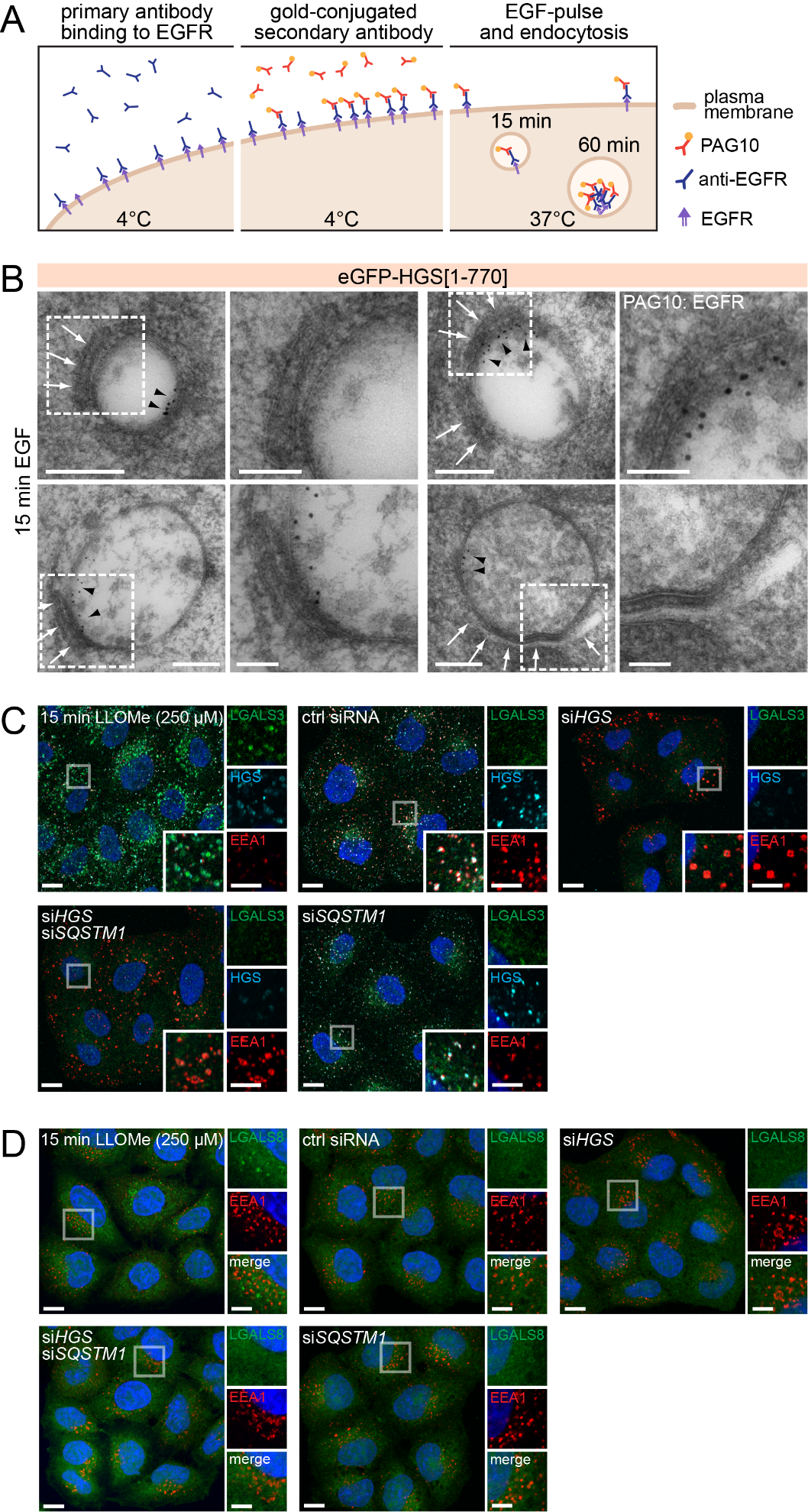


**Figure S2.** Simaphagy events occur independent of endosome/lysosome rupture. (**A**) Schematic representation of EGFR immunogold labelling method. Cell surface EGFR are detected by an anti-EGFR primary antibody. A secondary gold-conjugated antibody (PAG10) binds to the anti-EGFR antibody. The antibody labelling is performed at 4°C. Cells start EGFR internalization upon EGF-pulse and incubation at 37°C for 15 or 60 min. (**B**) Electron microscopy and immuno-gold labelling of EGFR in Hela cells expressing eGFP-HGS[1-770]. Endogenous HGS has been knocked down and cells were stimulated with EGF for 15 min. Black arrowheads highlight the PAG10-gold labelled EGFR receptors accumulating in a microdomain at the limiting membrane of endosomes. White arrows and insets indicate double membranes and a high electron density next to endosomes or the EGFR microdomain. Scale bar: 250 nm; 100 nm for insets. **(C)** Representative images from immunofluorescence staining of HeLa cells following 250 µM LLOMe treatment to induce endosome/lysosome damage, or after knockdown using siRNA against HGS, SQSTM1 or control siRNA. LGALS3 (galectin 3) labelling shows an increase upon 15 min LLOMe treatment, but not with HGS or SQSTM1 knockdown, indicating that no damaged endosomes are present. **(D)** Immunofluorescence labelling for LGALS8 (galectin 8) as described above. A LGALS8 recruitment to ruptured endosomes/lysosomes is detectable upon LLOMe treatment, but not upon siRNA knockdown of HGS or SQSTM1. Scale bar: 10 µm and 5 µm inset.


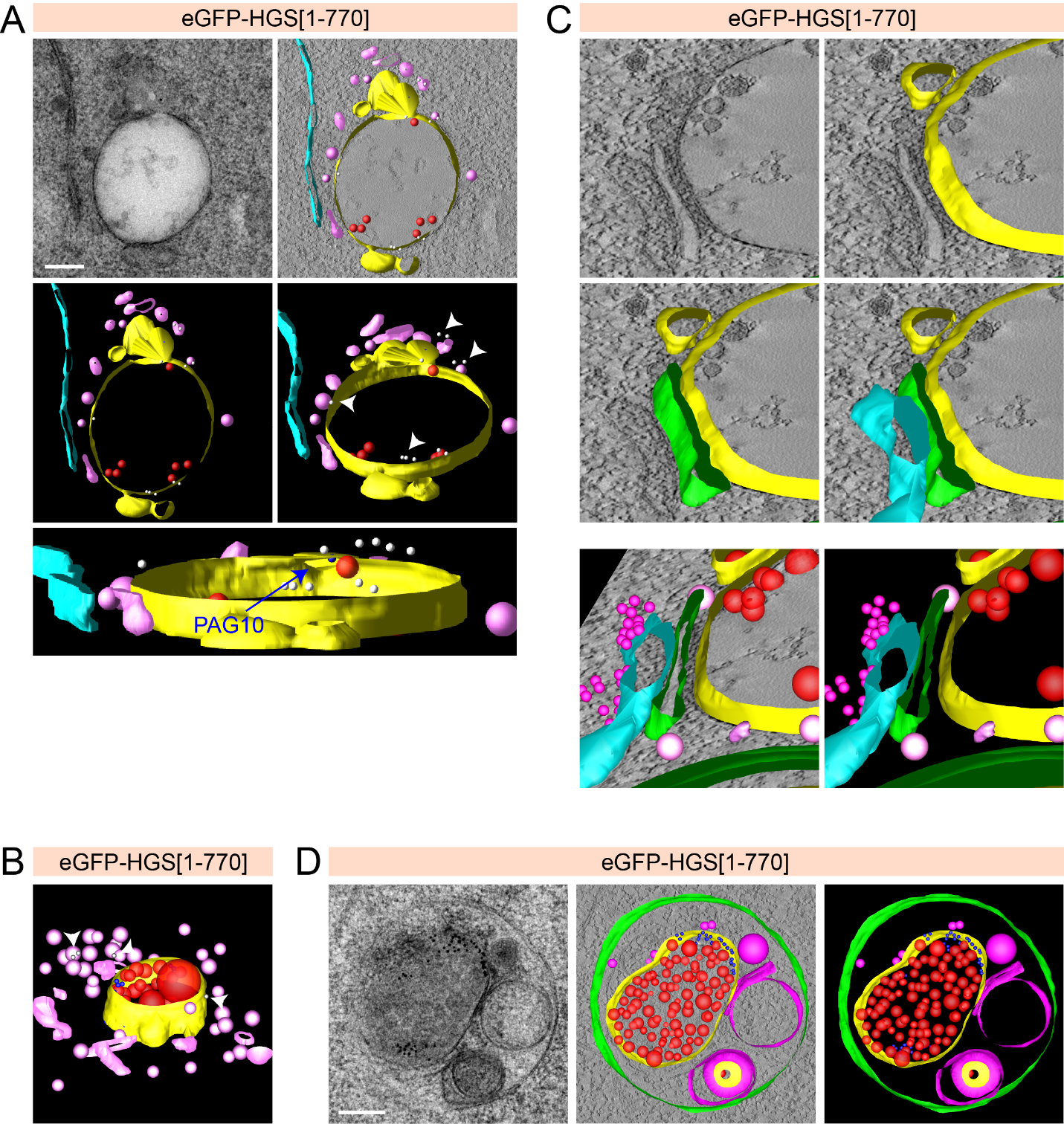


**Figure S3.** Ultrastructural analysis of simaphagy. (**A**) On-section immunogold-labelling with an antibody recognizing SQSTM1 (white dots and white arrowheads) can be seen in areas next to PAG10-containing (blue, in endosome protrusion) endosomes (yellow, ILVs in red). Tomograms reveal the presence of vesicles (light pink) below the sites of SQSTM1-labeling. Occasionally, also ER (cyan) can be found in proximity to such endosomes. Scale bar: 200 nm. (**B**) Endosome with on-section immunogold-labelling against SQSTM1 (white dots and white arrowheads) as shown in **Figure 2B**. The model is rotated 55 degrees along the X-axis. (**C**) Zoomed in area of endosome shown in **Figure 2C**. One can see a dark, electron-dense area between the endosome limiting membrane (yellow) and the double-membrane sheet (green) that runs parallel to the endosome. Portions of rough ER (cyan, ribosomes in pink) run adjacent, and parallel to this double membrane sheet. In the lower two panels, the model has been rotated along the X- and the Y-axis in order to give a better view of the three membranes (endosome, double membrane sheet, ER) running in parallel to each other. (**D**) Single membrane (green) surrounding a PAG10-containing endosome (yellow, ILVs in red) and portions of the cytosol/other organelles (pink) resembling an auto-lysosome in which the inner autophagic membrane has been degraded. Scale bar: 200 nm.


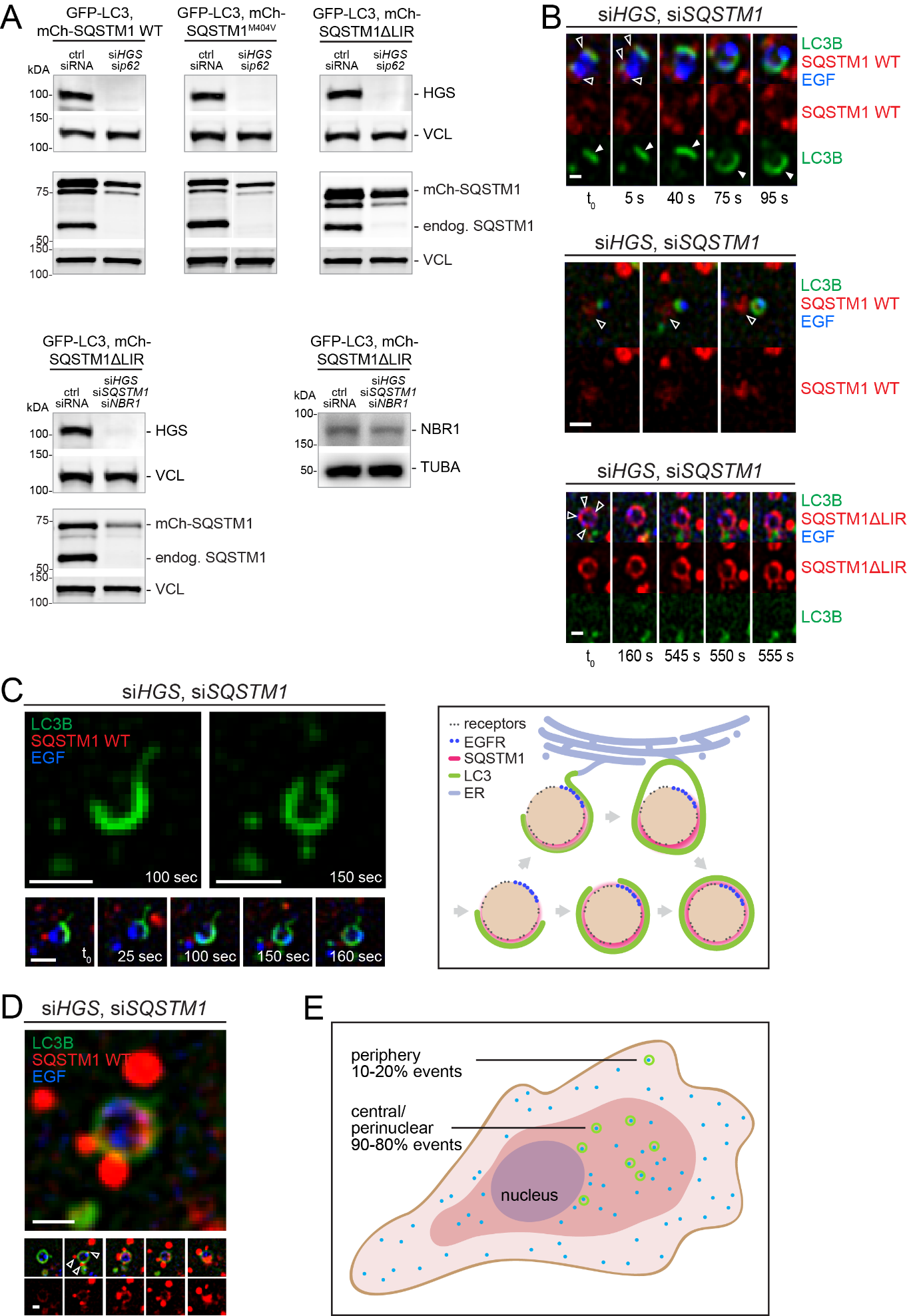


**Figure S4.** SQSTM1 is found in proximity to endosomes during initiation and later coats endosomes together with LC3B. (**A**) Western blots showing the knockdown of endogenous HGS, SQSTM1 and NBR1 in the stable RPE-1 cell lines used for the live-cell imaging experiments in **Figures 3, S5 and S6**. mCherry-SQSTM1 WT, -SQSTM1ΔLIR and -SQSTM1^M404V^ are siRNA resistant. (**B**) Representative recruitment-phase of SQSTM1 and LC3B to an endosome in RPE-1 stably expressing mCherry-SQSTM1 WT, GFP-LC3B. During the initiation phase, SQSTM1 WT is found on several spots in proximity to endosomes (empty arrowhead; left and middle panel). Upper panel: LC3B is recruited to only one location, and the phagophore extends from there (white arrowhead). Lower and middle panel: SQSTM1ΔLIR coats endosomes and displays domains with stronger accumulation. Here, GFP-LC3B fails to be recruited. Scale bar: 1 µm. (**C**) Representative movie stills from live-cell imaging of a growing GFP-LC3B positive phagophore membrane with a hook-shaped extension. The extension deforms dynamically and is likely connected to the adjacent ER network. A potential visualization of the phagophore – ER connection is shown in the scheme. Scale bar: 1 µm. (**D**) Representative movie stills showing lysosome recruitment and fusion after completion of simaphagy in RPE1 mCherry-SQSTM1 WT, GFP-LC3B cells. Lysosomes are indirectly visualized by mCherry-SQSTM1 fluorescence. While SQSTM1 is degraded in lysosomes, the mCherry protein remains stable and fluorescent [67 – 68], thus lysosomes become visible. Endogenous HGS and SQSTM1 are knocked down by siRNA. Arrowheads highlight docking lysosomes. Scale bar: 1 µm. (**E**) Schematic representation of relative occurrence and location of simaphagy events in HGS depleted cells as quantified from live cell imaging.


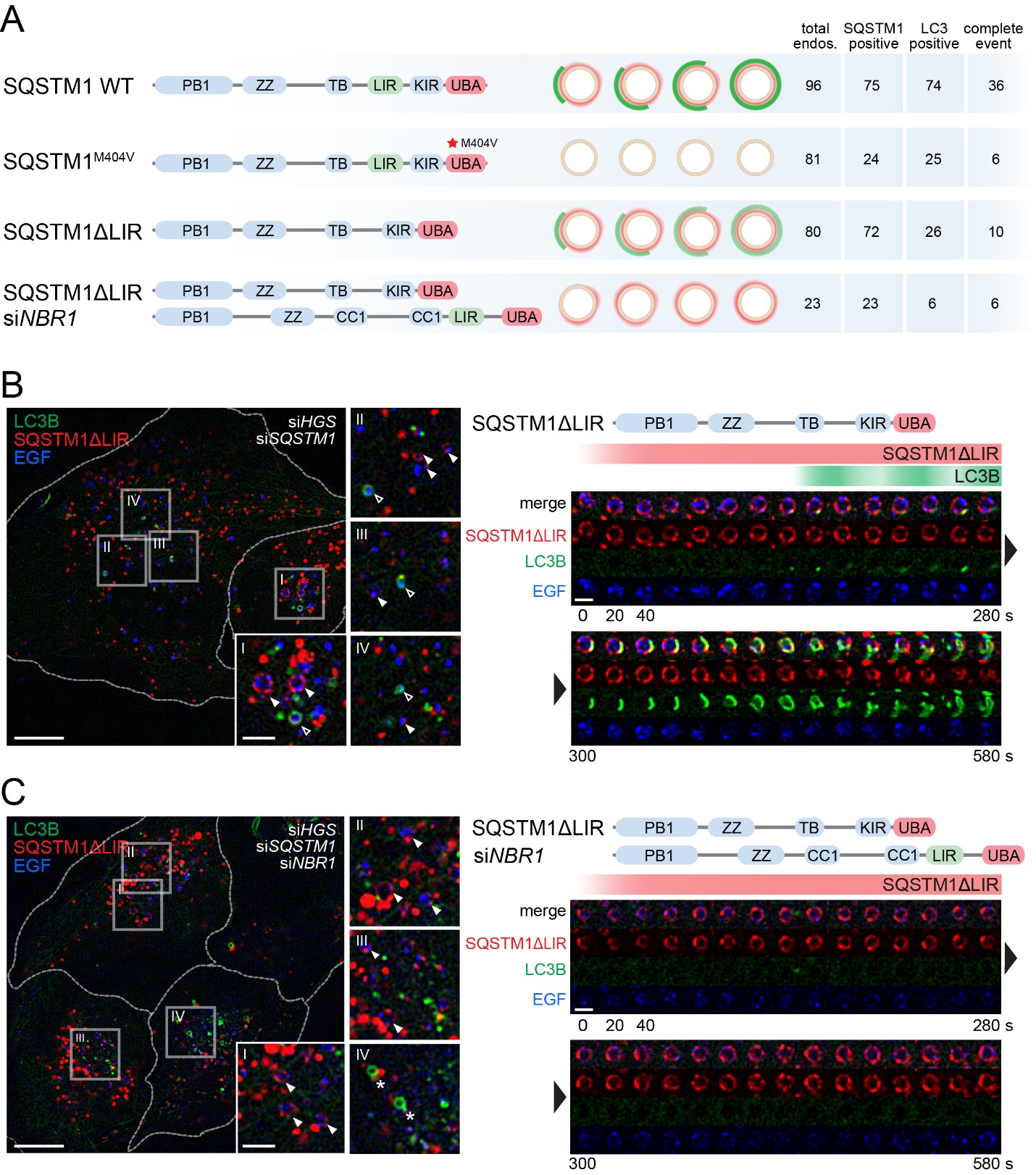


**Figure S5.** Both SQSTM1 and NBR1 are recruited to initiate successful simaphagy events. (**A**) Overview on the NBR1 and SQSTM1 domain structure and the different mutants of SQSTM1 used in the experimental setups. Next to it are schematic recruitment behaviors of LC3B and the different SQSTM1 mutants during simaphagy events. PB1 (SQSTM1 homo- and heterodimerization), ZZ (recognizes N′-end degrons), TB (TRAF6-binding), LIR (LC3 interacting region), KIR (Keap-1 interacting region), UBA (ubiquitin binding), CC1 (self-oligomerization). (**B**) Left: Movie stills from live-cell imaging as shown above using RPE-1 cells stably expressing GFP-LC3B and mCherry-SQSTM1ΔLIR. White arrowheads: simaphagy events with SQSTM1ΔLIR and LC3B recruitment. Hollow arrowheads: endosomes with exclusive SQSTM1ΔLIR, but no LC3B recruitment. Right: Domain structure of SQSTM1ΔLIR with deleted LIR-domain is shown, together with the timeline of a representative simaphagy attempt. The dynamic recruitment of SQSTM1ΔLIR and LC3B are shown by the red and green gradient respectively. Cell outlines shown by dotted line. Scale bar: 10 µm; 3 µm for insets I-IV; 2 µm for timeline. (**C**) Left: Representative movie stills from live-cell imaging using RPE-1 cells stably expressing GFP-LC3B and mCherry-SQSTM1ΔLIR. Endogenous HGS, SQSTM1 and NBR1 are knocked down. Insets I-IV: White arrowheads highlight endosomes with mCherry-SQSTM1ΔLIR only. Asterisks: non-endosome containing phagophores. Right: Domain structures of SQSTM1ΔLIR and NBR1. The recruitment dynamic of SQSTM1 is shown by the red gradient and the timeline of a representative endosome track. No LC3B is recruited. Cell outlines shown by dotted line. Scale bar: 10 µm; 3 µm for insets I-IV; 2 µm for timeline.

**
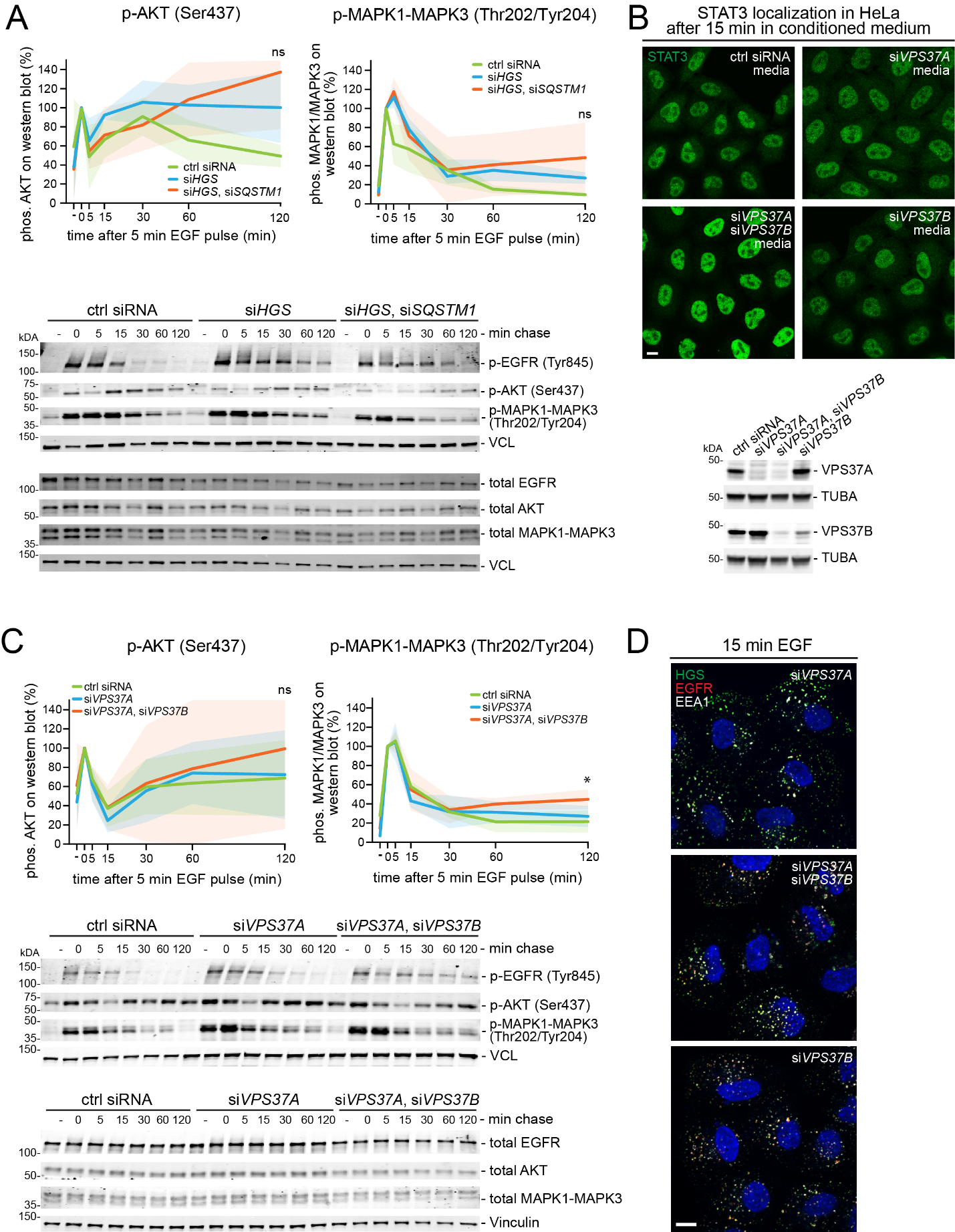
**

**Figure S6.** Loss of ESCRT and autophagy function sustains EGFR phosphorylation and downstream signaling. (**A**) Quantitative western blot analysis of AKT (Ser437) and MAPK1-MAPK3 (Thr202/Tyr204) in cells with HGS and SQSTM1 knockdown. Cells were subjected to a 5 min pulse with low dose EGF (5 ng/ml). Knockdown of HGS shows a trend for elevated AKT (Ser437) and MAPK1-MAPK3 (Thr202/Tyr204) phosphorylation. This is further increased by an additional knockdown of SQSTM1. Quantitation of AKT (Ser437) and MAPK1-MAPK3 (Thr202/Tyr204) amount on western blot are normalized to the loading control and shown in percent. t = 0 min chase was set to 100%. EGFR (Tyr845) is shown in **Figure 5A**. Results are mean ± SD of three independent experiments; One-way ANOVA, Tukey’s Test for multiple comparisons found no statistically significant difference for AKT (Ser437) and MAPK1-MAPK3 (Thr202/Tyr204) when comparing the mean values for 120 min between the groups [ctrl siRNA], [si*HGS*] and [si*HGS*, si*SQSTM1*] (ns= not statistically significant). (**B**) Representative immunofluorescence staining of HeLa cells after incubation with conditioned medium (15 min) derived from cells with the indicated knockdown of si*VPS37A* and/or si*VPS37B*. Scale bar: 10 µm. (**C**) Quantitative western blot analysis of AKT (Ser437) and MAPK1-MAPK3 (Thr202/Tyr204) in HeLa cells with knockdown of VPS37A and VPS37B. Cells were treated as described above. Knockdown of VPS37A alone had a mild effect on AKT (Ser437) and MAPK1-MAPK3 (Thr202/Tyr204) phosphorylation. Cells with VPS37A and VPS37B double knockdown show a trend for sustained AKT (Ser437) and MAPK1-MAPK3 (Thr202/Tyr204) phosphorylation. Quantitation of AKT (Ser437) and MAPK1-MAPK3 (Thr202/Tyr204) amount on western blot are normalized to the loading control and shown in percent. t = 0 min chase was set to 100%. EGFR (Tyr845) is shown in **Figure 5B**. Results are mean ± SD of three independent experiments; One-way ANOVA, Tukey’s Test for multiple comparisons identified a statistically significant difference for MAPK1-MAPK3 (Thr202/Tyr204) mean values at 120 min when comparing [ctrl siRNA] and [si*VPS37A*, si*VPS37B*]. When comparing AKT (Ser437) mean values for 120 min we found no statistically significant difference between [ctrl siRNA], [si*VPS37A*] and [si*VPS37A*, si*VPS37B*] (*p < 0.1, ns = not statistically significant). (**D**) Representative immunofluorescence staining of cells with VPS37A and VPS37B knockdown 15 min after an EGF stimulation. Extension to **Figure 5C**.


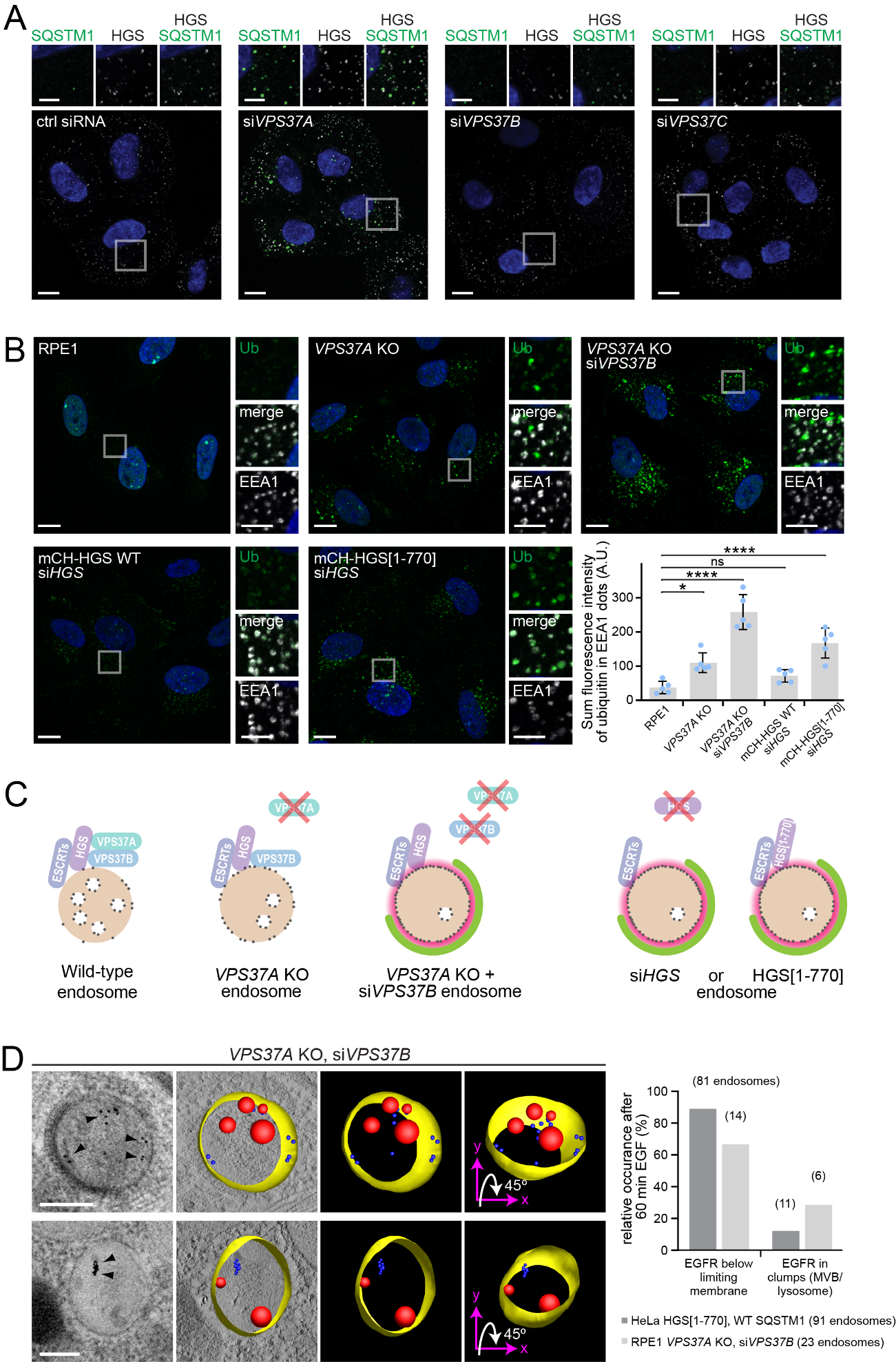


**Figure S7.** VPS37A and VPS37B co-depletion leads to increased receptor accumulation. (**A**) Representative immunofluorescence of HeLa cells subjected to siRNA knockdown of VPS37 isoforms and stained for HGS and SQSTM1. Knockdown of VPS37A, but not VPS37B or VPS37C, leads to an increase in SQSTM1 positive structures. Scale bar: 10 µm; 5 µm for insets. (**B**) Representative immunofluorescence staining in RPE-1 cells and quantification of ubiquitinated receptors (Ub) on EEA1 positive endosomes. *VPS37A* KO cells show an increase of ubiquitin on endosomes compared to control cells. This is enhanced upon additional knockdown of VPS37B. mCherry-HGS WT manages to rescue the knockdown of endogenous HGS and ubiquitinated receptors are degraded, while mCherry-HGS[1-770] cells strongly accumulate ubiquitin. Quantification from one staining shown, same results observed in other independent experiments. 50 - 60 cells per condition were quantified. Mean ± SD are shown; One-way ANOVA *p < 0.1, ****p <0.0001, ns = not statistically significant. Scale bar: 10 µm; 5 µm for insets. (**C**) Schematic summary of normal receptor degradation, accumulation and simaphagy events. Loss of VPS37A leads to an accumulation of receptors on endosomes, but possibly not enough to trigger SQSTM1 and LC3B recruitment. Co-depletion of VPS37A and VPS37B potentially overcomes the threshold of ubiquitinated receptors and initiates simaphagy. HGS knockdown or expression of HGS[1-770] mutant accumulate sufficient ubiquitinated receptors to trigger simaphagy. (**D**) Electron tomogram and 3D-model of representative endosomes in *VPS37A* KO cells with a VPS37B knockdown. Immuno-gold labelled EGFR is highlighted (black arrowheads) after 60 min EGF stimulation. Upper row: EGFR (blue) is found adjacent to the limiting membrane (yellow); ILVs (red). Lower row: EGFR clumped together inside the endosome. Quantified relative occurrence of EGFR below the limiting membrane and in clumps in (MVBs/lysosomes) is shown for the indicated cell lines. Tomograms of 250-nm sections. Scale bar: 200 nm.

**
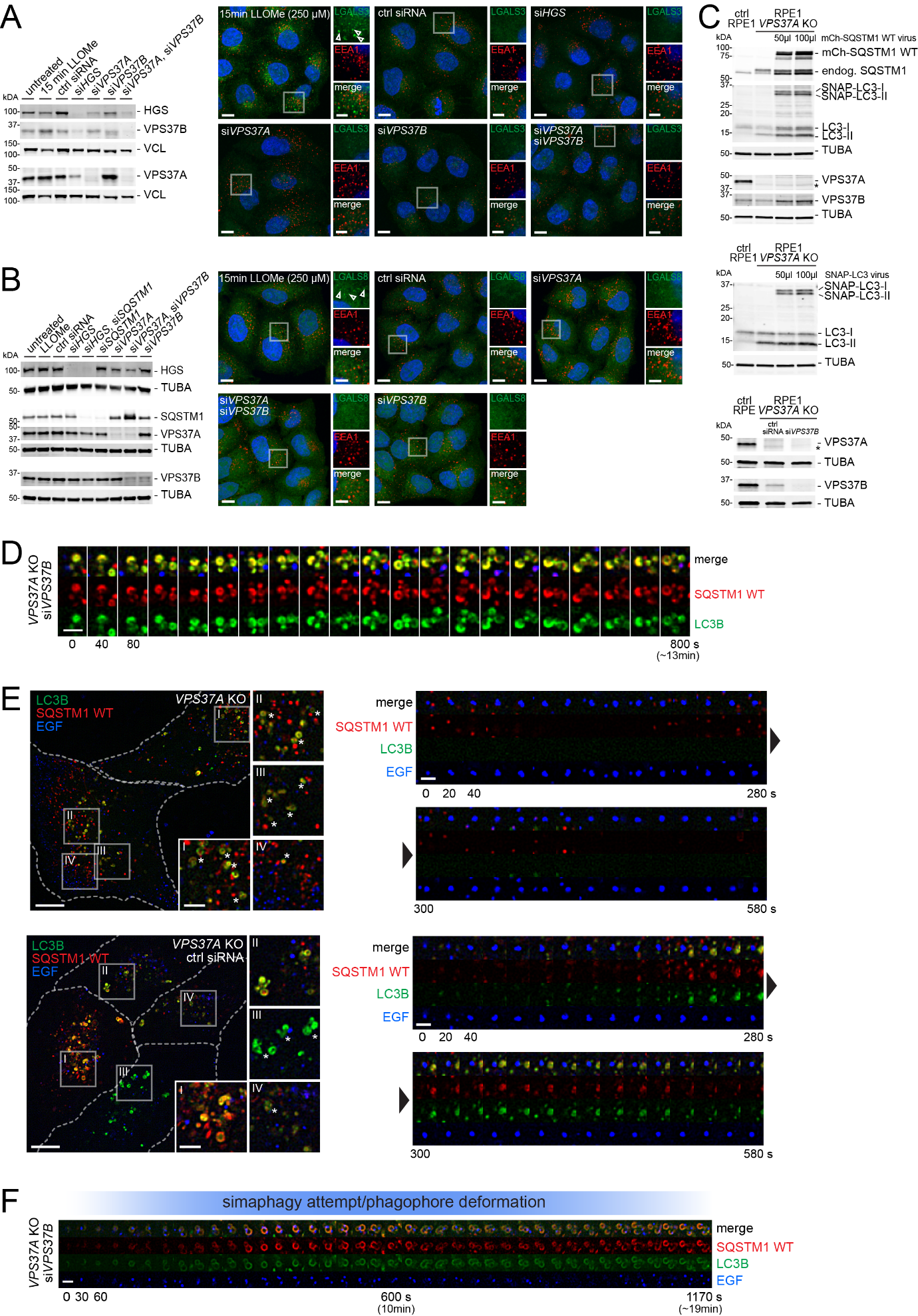
Figure S8.** Receptor accumulation and not endosome rupture initiates simaphagy. (**A**) Representative images from immunofluorescence staining in HeLa cells following 250 µM LLOMe treatment and induction of endosome/lysosome damage. LGALS3 staining is strongly visible after 15 min LLOMe. Knockdown of VPS37A and VPS37B or in combination, did not increase the number of LGALS3 labelled endosomes. Equally, no endosome rupture is detectable in HGS depleted or control cells. Scale bar: 10 µm; 5 µm for insets. Western blot of cell lysates from this experiment is shown. (**B**) Representative immunofluorescence labelling for LGALS8. 250 µM LLOMe induces a visible LGALS8 recruitment, which is not the case for siRNA treated cells. Scale bar: 10 µm; 5 µm for insets. Western blot of cell lysates from this experiment and LGALS8 labelling in **Figure S2** is shown. (**C**) Left: Representative western blot of RPE-1 control and *VPS37A* KO cells, including knockdown efficiency of VPS37B using siRNA. VPS37A antibody with unspecific background band (asterisk). Middle: *VPS37A* KO leads to a lipidation of LC3B and oligomerization of SQSTM1. Right: Generation of stable cell lines expressing SNAP-LC3B and mCherry-SQSTM1 WT. Cells with low or close to the endogenous expression levels were chosen for imaging experiments. (**D**) Representative movie stills from RPE-1 *VPS37A* KO cells with a knockdown of VPS37B showing a cluster of phagophores (not containing endosomes). The phagophore membranes are dynamically deforming, budding and opening over time. Scale bar: 2 µm. (**E**) Movie stills from live-cell imaging of RPE-1 *VPS37A* KO cells, stably expressing SNAP-LC3B and mCherry-SQSTM1 WT. Cells were stimulated with a 2 min EGF-Alexa Fluor 647 pulse. No simaphagy events were detected. Cells display an accumulation of SQSTM1 and LC3B positive phagophores (asterisks), which are not related to endosomes as judged by absence of EGF signal. Timeline of a representative endosome labelled with EGF-Alexa Fluor 647 and no visible recruitment of SQSTM1 or LC3B. Cells transfected with control siRNA display the same phenotype. Scale bar: 10 µm; 3 µm for insets I-IV; 2 µm for timeline. (**F**) Timeline of a representative simaphagy event in RPE-1 *VPS37A* KO cells with a VPS37B knockdown. EGF-Alexa Fluor 647 positive endosomes are recognized by mCherry-SQSTM1 WT and SNAP-LC3B and engulfed in a phagophore. These simaphagy events are stalled or phagophores open or deform during the observation time. No recruitment and fusion of lysosomes was observed. Scale bar: 2 µm.
