# Supplementary figures and images for: Removal of hypersignaling endosomes by simaphagy
Source: Autophagy. 2023 Oct 16;20(4):769–91. doi: 10.1080/15548627.2023.2267958 (PMC11062362; doi:10.1080/15548627.2023.2267958)

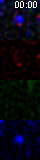

Supplement: Supplemental Material [file KAUP_A_2267958_SM7544.zip › Movie_1 (1).tif]

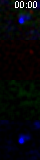

Supplement: Supplemental Material [file KAUP_A_2267958_SM7544.zip › Movie_2.tif]

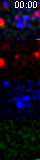

Supplement: Supplemental Material [file KAUP_A_2267958_SM7544.zip › Movie_3.tif]

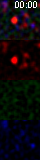

Supplement: Supplemental Material [file KAUP_A_2267958_SM7544.zip › Movie_4.tif]

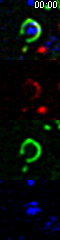

Supplement: Supplemental Material [file KAUP_A_2267958_SM7544.zip › Movie_5.tif]

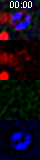

Supplement: Supplemental Material [file KAUP_A_2267958_SM7544.zip › Movie_6.tif]

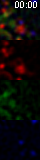

Supplement: Supplemental Material [file KAUP_A_2267958_SM7544.zip › Movie_7.tif]

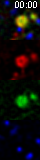

Supplement: Supplemental Material [file KAUP_A_2267958_SM7544.zip › Movie_8.tif]
